# Modeling the dynamics of COVID-19 with real data from Thailand

**DOI:** 10.1038/s41598-023-39798-9

**Published:** 2023-08-11

**Authors:** Alhassan Ibrahim, Usa Wannasingha Humphries, Parinya Sa Ngiamsunthorn, Isa Abdullahi Baba, Sania Qureshi, Amir Khan

**Affiliations:** 1 Department of Mathematics, Faculty of Science, King Mongkut’s University of Technology, Thonburi (KMUTT), 126 Pracha Uthit Road, Bang Mod, Thung Khru, Bangkok, 10140 Thailand; 2 Department of Mathematical Sciences, Bayero University, Kano, Nigeria; 3 Department of mathematics, Near East University TRNC, Mersin 10, Turkey; 4 Department of Basic Sciences and Related Studies, Mehran University of Engineering & Technology, Jamshoro, 76062 Pakistan; 5 Department of Mathematics and Statistics, University of Swat, Khyber Pakhtunkhwa, kpk Pakistan

**Keywords:** Applied mathematics, Scientific data

## Abstract

In recent years, COVID-19 has evolved into many variants, posing new challenges for disease control and prevention. The Omicron variant, in particular, has been found to be highly contagious. In this study, we constructed and analyzed a mathematical model of COVID-19 transmission that incorporates vaccination and three different compartments of the infected population: asymptomatic $(I_{a})$, symptomatic $(I_{s})$, and Omicron $(I_{m})$. The model is formulated in the Caputo sense, which allows for fractional derivatives that capture the memory effects of the disease dynamics. We proved the existence and uniqueness of the solution of the model, obtained the effective reproduction number, showed that the model exhibits both endemic and disease-free equilibrium points, and showed that backward bifurcation can occur. Furthermore, we documented the effects of asymptomatic infected individuals on the disease transmission. We validated the model using real data from Thailand and found that vaccination alone is insufficient to completely eradicate the disease. We also found that Thailand must monitor asymptomatic individuals through stringent testing to halt and subsequently eradicate the disease. Our study provides novel insights into the behavior and impact of the Omicron variant and suggests possible strategies to mitigate its spread.

## Introduction

COVID-19 has been a global pandemic that has persisted for over three years, with no signs of slowing. The continuous mutation and evolution of the virus poses challenges in containing its spread^1^. In addition, social distancing and other mitigation measures are becoming increasingly difficult to maintain as people grow tired^2^. Therefore, it is likely that COVID-19 will remain a major concern in the near future, bearing major implications for both individuals and businesses alike. On an individual level, this means continuing to take precautions, such as wearing masks and avoiding large gatherings. This means that businesses must remain adaptable and undergo operational changes to remain viable.

The newly discovered COVID-19 variants are causing considerable concern. The so-called “Omicron” variant, which was first discovered in November 2021 in South Africa, was listed as a variant of concern (VOC) by November 26th^3^. This variant is thought to be more contagious than the original virus, and may also be more resistant to existing vaccines^4^. This is a major cause of concern as it could potentially lead to another wave of infection.

Thailand has suffered from COVID-19 since the outbreak of the pandemic^5^. During the last couple of years, the country has experienced a number of outbreaks, and owing to the emergence of new variants of this virus, the situation has been made even worsened^6^. Although the government has imposed strict measures to eliminate the threat, these measures have not been very effective in eradicating the virus^7^. Many people are now concerned about the possibility of further outbreaks owing to the current situation, and there is a great deal of public anxiety regarding the situation.

Although COVID-19 drugs are not currently available^8^, there are a number of vaccines available that can help prevent COVID-19 infection. The first one is the RNA-based (mRNA) vaccines which have some advantages over conventional vaccines. The mRNA vaccines are not infectious since they are not created using pathogen particles or inactivated pathogens, unlike the conventional vaccines that rely on the production of pathogens, which can cause outbreaks of the disease if done in enormous quantities^9^. Some representatives of the RNA-based vaccines are reported as *Moderna COVID-19 vaccine* or *mRNA-1273*^10^, *Comirnaty* or *BNT162b2*^11^, and *CVnCoV*^12^.

Next is the DNA vaccine which is a new type of vaccine that uses DNA plasmids to trigger an immune response in the host, this allows the body to create its own immunity against the disease. DNA vaccines are often produced rapidly and at a low cost, which is one of the advantages of using them^13^. Additionally, there is no chance that this vaccine causes infection. Some representative of the DNA vaccines are *AG0301-COVID19*^14^ and *Covigenix VAX-001*^15^.

Another form of COVID-19 vaccine is the viral vector vaccine which allows a weakened form of the virus to be used in vaccine development^16^. There are several factors that hinder the efficacy of this vaccine, including the pre-existing immunity of the host^17^. *Sputnik V*, *Janssen COVID-19 vaccine*, and *COVID-19 vaccine AstraZeneca*, are some representatives of viral vector vaccines. These vaccines have various levels of efficacy associated with them^18^.

In November 2022, World Health Organization (WHO) reported that at least one dose of the COVID-19 vaccine was given to a total of 57, 376, 849 people, and about 53, 923, 816 have been fully vaccinated in Thailand^19^. The country’s vaccination program began in February 2021, using Sinovac and AstraZeneca vaccines^20^. With this measure, Thailand was a step closer to achieving herd immunity against this virus, which is essential to the prevention of any future outbreaks. However, some challenges occur that slow the process, firstly, there was a vaccine shortage in the country. As a result, many people are unable to get vaccinated, hampering the efforts to vaccinate as many people as possible^21^. The second problem is that there is a lack of awareness about the importance of vaccination and how to get it^22^. Due to misinformation and conspiracy theories regarding the vaccine’s effectiveness and safety, there are still a lot of people who are hesitant to get the vaccine.

To understand the method of propagation of COVID-19 and in order to contribute to its eradication, many researchers developed mathematical models in the literature (see for instance^23–25^). The COVID-19 infection has several unique characteristics that make it difficult to control. Firstly, the COVID-19 asymptomatic infected individuals, and the disease incubation period, which is two to fourteen days, implies that individuals can be infected with COVID-19 and infect others before they even realize they have it. Secondly, COVID-19 resembles other respiratory illnesses in terms of symptoms, making it very difficult to diagnose. Due to these factors, numerous models that consider the symptomatic and asymptomatic infected population have been developed, and some other models considered control measures like testing, face masks, etc. See the following papers and the references therein^26–30^.

Photphanloet et al. in^31^ considered a COVID-19 epidemic model using nonlinear ordinary differential equations. The model divides the Thailand population into 7 compartments that are used for predicting the potential effect of non-pharmaceutical interventions and vaccination in the transmission dynamics of COVID-19. Vaccination, however, was shown to play a critical role in halting the virus spread, and that non-pharmaceutical interventions, like wearing recommended face masks, social distancing, and hand washing, can also help in reducing the transmission. The authors neglected the asymptomatic individuals which pose a significant challenge in the infection control of COVID-19. Some other features of COVID-19 that are not considered are the immunity development by vaccinated individuals and the immunity loss by the recovered people. It has been observed that this phenomenon occurs in many epidemic models as well (see, for instance^26,27,32^,).

COVID-19 is a disease with partial immunity; after recovery, one can get reinfected. Hence, memory plays a vital role in the study of disease dynamics^33^. Fractional-order differential equations can be used to model systems using memory^34^. This allows for a more accurate modeling of the system because the current state of a system is influenced not only by the immediate past but also by the distant past, which makes it possible to better predict its future. Fractional differential equations are used to improve the accuracy of epidemiological predictions^35^. For example, Khan and Atangana^26^ considered a fractional model with six compartments susceptible (*S*), exposed (*E*), asymptomatic infected $(I_{a})$, symptomatic infected $(I_{s})$, omicron-infected $(I_{o})$, and recovered (*R*) individuals to analyze the dynamics of COVID-19 using Omicron features. An analysis of the fractional model and numerical simulations are presented. In the course of their research, they were able to show that those infected with omicrons and asymptomatic individuals can spread the infection and develop further infections in South Africa if they encounter healthy individuals.

The COVID-19 pandemic has posed a serious threat to public health and socio-economic stability worldwide. In Thailand, the situation has been exacerbated by the emergence of new variants, such as the Omicron variant, which have increased the transmissibility and severity of the disease. To understand and control the spread of the virus, mathematical models can provide useful insights and predictions based on available data and assumptions. However, most existing models do not account for some important factors that may affect the dynamics of the infection, such as the role of asymptomatic individuals, the immune response, and the impact of vaccination. Therefore, in this study, we aim to fill this gap by developing a fractional-order model that incorporates these factors and captures the essential features of the COVID-19 epidemic in Thailand. Our model is motivated by the work of Photphanloet et al.^31^, who proposed a fractional-order model for COVID-19 with memory and an asymptomatic population. We extended their model by adding a vaccine compartment and allowing transitions from the asymptomatic compartment to either the symptomatic or Omicron compartment. We also consider the possibility of Omicron-induced death in our model. By doing so, we hope to provide a more realistic and comprehensive description of the COVID-19 situation in Thailand and offer some useful recommendations for disease control and prevention.

As a result of this paper, the following conclusions can be drawn:

1. We propose an $S V E I_{s} I_{a} I_{m} R$ model by including a parameter that represents immunity development in vaccinated individuals. This will help determine how well the vaccine works and help in the control of COVID-19 spread.
2. The model is further extended to fractional order in the Caputo sense.
3. Theoretical results are established
4. A global sensitivity analysis was performed.
5. Numerical illustrations were also performed.

The fractional-order framework for conceptualizing COVID-19 offers several epidemiological advantages over classical integer-order models. The implementation offers several epidemiological advantages. First, it acknowledges the intricate dynamics of the virus, including super-spreading events, variable transmission rates, and the occurrence of multiple waves of infection^36,37^. Second, fractional-order models capture the persistence of memory, accounting for the past trajectory of an epidemic, thereby providing a more accurate representation of its dynamics^38–44^. These models also enable the evaluation of intervention effectiveness by considering the time-dependent impact of measures, such as NPIs and vaccination campaigns. Moreover, fractional-order models enable multiscale analysis, capturing interactions at different levels, which enhances their reliability for informed decision-making in public health interventions and resource allocation^45^.

The remainder of this paper is arranged as follows: “Model formulation” section develops our model and provides mathematical preliminaries, as well as extending the model to fractional order. “Qualitative properties of the model” section presents a qualitative analysis of the proposed model, including its stability and bifurcation properties. “Numerical simulations of the model” section conducts numerical simulations of the Caputo fractional-order model and performs global sensitivity analysis to identify the key parameters affecting the dynamics. “Discussion” section discusses the results and their implications for disease control and prevention. “Conclusions” section concludes the paper with some remarks and future directions.

## Model formulation

Here, we present a compartmental model that studies the spread of coronavirus in Thailand. The model is constructed as follows: we divide the infected class into three sub-classes $I_{a}, I_{s}, I_{m}$ that are asymptomatic infected, symptomatic infected (infected individuals showing clinical symptoms of COVID-19 most commonly: fever or chills, cough, shortness of breath, sore throat, loss of taste or smell), and infected individuals showing clinical symptoms unique to omicrons (sore throat, particularly a ”scratchy” throat, persistent cough^46^). Thus, the total population *N* is defined as

1 $$\begin{aligned} N = S + V + E + I_{s} + I_{a} + I_{m} + R. \end{aligned}$$

At rate $\Lambda$, susceptible individuals are recruited into the population, and recovered individuals lose immunity and move to this population at rate $\rho$. When certain people receive vaccination, the population decreases at rate $\omega (0\!<\omega \le 1)$. This population further decreases when some individuals are exposed to the force of infection, as follows:

2 $$\begin{aligned} \eta _{s} = \frac{\beta (I_{a} + \nu _{1} I_{s} + \nu _{2} I_{m})}{N}, \end{aligned}$$

where $\nu _{1}$ and $\nu _{2}$ are the probability of infectiousness of $I_{s}$ and $I_{m}$ respectively, and $\beta$ is the effective contact rate.

$\tau$ indicates the rate of incubation of the exposed individuals that are infected with the virus without showing symptoms at the rate $\varpi$, showing symptoms of other variants at the rate $\sigma$ or showing unique symptoms of Omicron at a rate of $(1 - \sigma - \varpi )$. Parameters $\alpha _{1}, \alpha _{2} \text { and } \alpha _{3}$ provide information on the recovery of asymptomatic, symptomatic, and omicron-infected individuals, respectively. $\delta _{1} \text { and } \delta _{2}$ are the rates at which symptomatic people and those in the omicron class die due to the disease, respectively.

**Figure 1:**
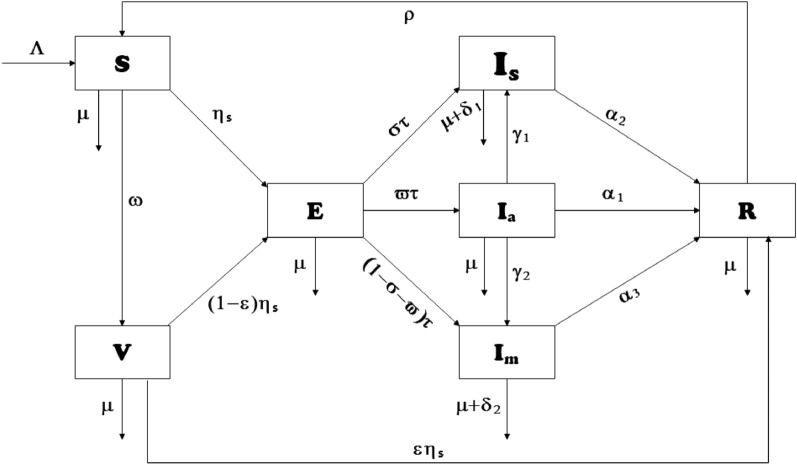
Schematic diagram of (3).

Figure 1 depicts the schematic diagram of the model, whereas Tables 1 and 2 provide the meanings of the variables and parameters, respectively.

3 $$\begin{aligned} \left\{ \begin{aligned}&\frac{dS}{dt} = \Lambda + \rho R - (\eta _{s} + \omega + \mu )S, \\&\frac{dV}{dt} = \omega S - (\eta _{s} + \mu )V, \\&\frac{dE}{dt} = \eta _{s} S + (1 - \epsilon )\eta _{s} V - (\tau + \mu )E, \\&\frac{dI_{s}}{dt} = \sigma \tau E + \gamma _{1} I_{a} - (\alpha _{2} + \delta _{1} + \mu )I_{s}, \\&\frac{dI_{a}}{dt} = \varpi \tau E - (\gamma _{1} + \gamma _{2} + \alpha _{1} + \mu )I_{a}, \\&\frac{dI_{m}}{dt} = (1 - \sigma - \varpi )\tau E + \gamma _{2} I_{a} - (\alpha _{3} + \delta _{2} + \mu )I_{m}, \\&\frac{dR}{dt} = \epsilon \eta _{s} V + \alpha _{1} I_{a} + \alpha _{2} I_{s} + \alpha _{3} I_{m} - (\rho + \mu ) R, \end{aligned}\right. \end{aligned}$$

**Table 1:** Meaning of variables.

Variable	Meaning
*S*	Susceptible individuals
*V*	Vaccinated individuals
*E*	Exposed individuals
$I_{a}$	Asymptomatic infected individuals
$I_{s}$	Symptomatic infected individuals
$I_{m}$	Individuals with omicron symptoms
*R*	Recovered individuals

**Table 2:** Meanings of parameters and their units.

Parameter	Meaning and units
$\Lambda$	Recruitment rate (day$^{-1}$)
$\beta$	Effective contact rate (per person $\cdot \text { day } ^{-1}$)
$\omega$	Vaccination rate (day$^{-1}$)
$\epsilon$	Infection reduction of vaccinated individuals (day$^{-1}$)
$\nu _{1}$	Probability of infectiousness of symptomatic individuals $I_{s}$
$\nu _{2}$	Probability of infectiousness of omicron infected individuals $I_{m}$
$\mu$	Natural death rate (day$^{-1}$)
$\tau$	Disease incubation period (day$^{-1}$)
$\varpi$	Rate of progression from exposed individuals to asymptomatic infected individuals (person $\cdot \text { day } ^{-1}$)
$\sigma$	Rate of progression from exposed individuals to symptomatic infected individuals (person $\cdot \text { day } ^{-1}$)
$\gamma _{1}$	Rate at which asymptomatic infected person start showing symptoms of other variants (person $\cdot \text { day } ^{-1}$)
$\gamma _{2}$	Rate at which asymptomatic infected start showing symptoms attributed to omicron (person $\cdot \text { day } ^{-1}$)
$\delta _{1}$	COVID-19 induced mortality rate on $I_{s}$ (day$^{-1}$)
$\delta _{2}$	COVID-19 Omicron induced mortality rate on $I_{m}$ (day$^{-1}$)
$\alpha _{1}, \alpha _{2}, \alpha _{3}$	Rate of recovery of $I_{a}, I_{s} \text { and } I_{m}$ respectively (day$^{-1}$)
$\rho$	The rate at which recovered individuals lost immunity (day$^{-1}$)

### Parameter estimation and model fitting

Obtaining optimal parameter values and performing model validations are crucial when working with mathematical models that utilize real data. This is primarily because the accurate identification of parameter values from obtained data is often challenging. It is essential to obtain well-fitted parameter values for a specific model. Certain parameters associated with the epidemic can be computed by considering both the initial behavior of the epidemic and demographic factors linked to the disease. Additionally, the parameter values can be obtained from the existing literature and guided estimations. However, relying solely on this approach can occasionally result in erratic behavior.

To collect authentic and reliable cases from the population infected with COVID-19, it is essential to determine the appropriate biological characteristics that characterize these cases. These types of real-life examples can be offered for a period ranging from days to weeks to months to even years. Due to uncertainties in data analysis, there is a possibility that the conclusion could be inaccurate. Although there are numerous methods in the literature that can be used to estimate parameter values, the least-squares method is the most frequently employed. The method uses the idea of minimizing residuals between available infections for real data ${\bar{y}}_{j} = 0, 1, \ldots , n$ and the discrete points obtained with the suggested set of simulation equations $f(t_{j}, y_{j})$ as given below:

4 $$\begin{aligned} {\text {Residual}} = \dfrac{1}{N} \sum _{j=0}^{N} \Bigg | \dfrac{{\bar{y}}_j-y_j}{{\bar{y}}_j} \Bigg |, \end{aligned}$$

The aforementioned objective has been accomplished by utilizing the built-in routines of NonlinearModelFit and ParametricNDSolve that are included in the programming language known as Wolfram Mathematica 12.1. These fitted parameters are displayed in Table 3

**Figure 2:**
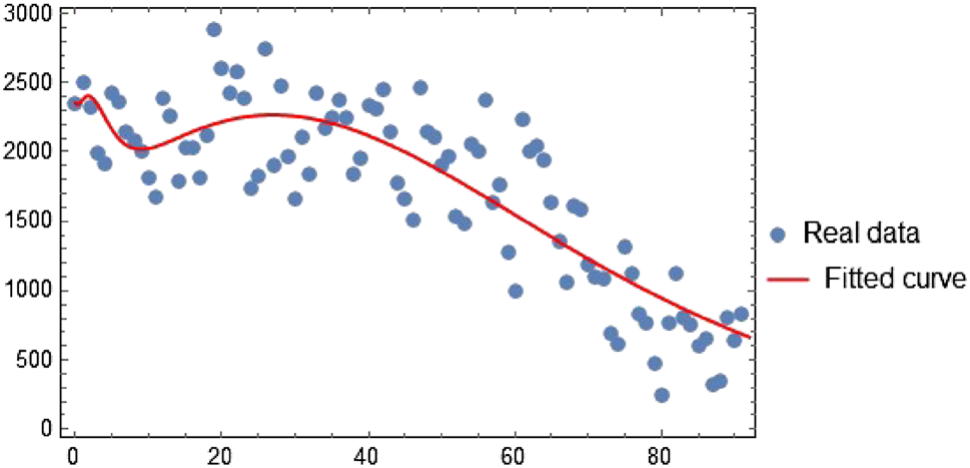
The curve fitting of model (3) simulations with the real cases of the disease.

**Figure 3:**
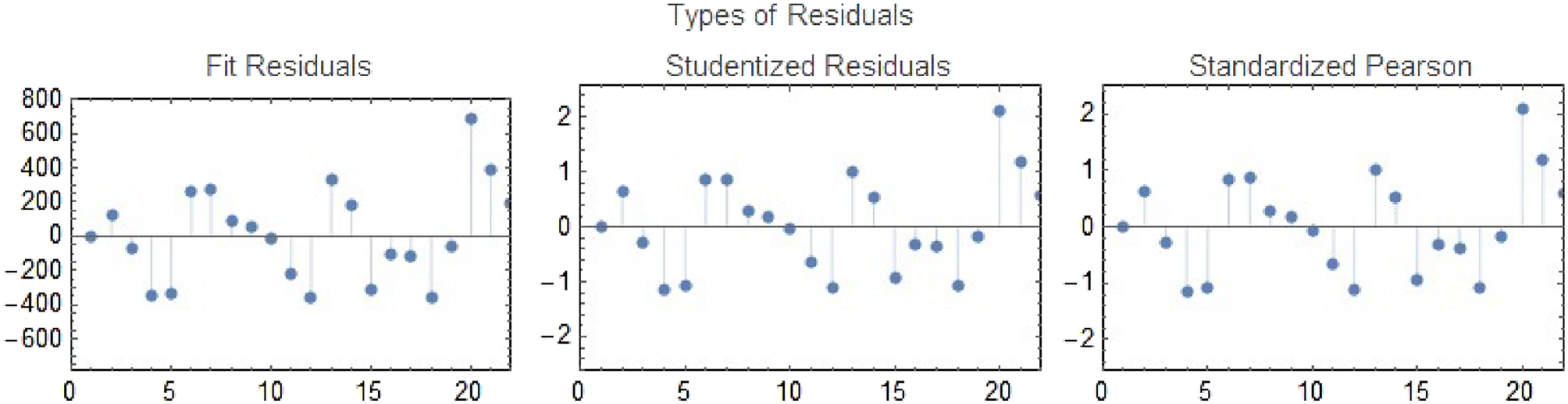
Various types of residuals for the curve fitting of (3) with the real cases of the disease.

Using real data from Thailand, which ranges from 1st July 2022 to 30th September 2022 (see^19,47^), the parameters of (3) were estimated. Thailand’s initial population was estimated to be $N(0) \simeq 70,000,000$^48,49^. The initial populations of reported vaccinated and infectious individuals are given by $V(0) = 107,912 \text {, and } I_{s}(0) + I_{m}(0) = 2354$ respectively. The initial susceptible population was $S(0) = 69,688,560$. We assume the initial population of the exposed, asymptomatic infected, and recovered individuals to be $E(0) = 100,000, I_{a}(0) = 1177 \text {, and } R(0) = 100,000$. With the help of these conditions and the parameters listed in Table 3, the fitted curve is obtained in Fig. 2 where the statistical $R^2$ value is computed as $\approx 0.97$ showing that the regression line perfectly fits the data. Moreover, the residuals in Fig. 3 are randomly distributed around a mean of zero, indicating a good fit.

**Table 3:** Parameters values.

Parameters	Value	Source
$\Lambda$	5	^28^
$\beta$	0.6886	^50^
$\omega$	0.0220	^51^
$\epsilon$	0.5000	^51^
$\nu _{1}$	0.0880	^51^
$\nu _{2}$	0.4074	Fitted
$\mu$	$\frac{1}{74.2 \times 365}$	^52^
$\tau$	0.8999	^26^
$\varpi$	0.8600	Estimated
$\sigma$	0.01216	Fitted
$\gamma _{1}$	0.1900	^53^
$\gamma _{2}$	0.1900	Estimated
$\delta _{1}$	0.5068	Fitted
$\delta _{2}$	0.0839	Fitted
$\alpha _{1}$	$\frac{1}{10}$	^54^
$\alpha _{2}$	0.0625	^55,56^
$\alpha _{3}$	0.1516	Fitted
$\rho$	1.5095	Fitted

### $S V E I_{s} I_{a} I_{m} R$ model in Caputo fractional operator form

In this section, we transform model (3) using the Caputo fractional derivatives in a similar manner^57^. First, we recall some preliminaries.

#### Definition 1

(See^58^) Let *g*(*t*) be a function that satisfies some smoothness condition and $\alpha > 0 \text { s.t } \alpha , t \in {\mathbb {R}}$, the derivative in Caputo form is define as:

5 $$\begin{aligned} ^{c}{\mathcal {D}}^{\alpha }_{t} g(t) = \frac{1}{\Gamma (n - \alpha )} \int _{0}^{t} (t - \xi )^{n - \alpha - 1} \frac{d^{n} g(\xi )}{d \xi ^{n}} d \xi , \text { where } n - 1 < \alpha , n \in {\mathbb {N}}. \end{aligned}$$

and

6 $$\begin{aligned} ^{c}{\mathcal {D}}^{\alpha }_{t} g(t) = \frac{1}{\Gamma (1 - \alpha )} \int _{0}^{t} (t - \xi )^{- \alpha } g'(\xi ) d \xi , \end{aligned}$$

for $n = 1$ and $\alpha \in (0,1]$. Also, $\alpha > 0$ the corresponding fractional integral is defined as

7 $$\begin{aligned} ^{c}{\mathcal {I}}^{\alpha }_{t} g(t) = \frac{1}{\Gamma (\alpha )} \int _{0}^{t} (t - \xi )^{ \alpha - 1} g(\xi ) d \xi . \end{aligned}$$

#### Definition 2

[Mittag-Leffler^59^] This is defined as:

8 $$\begin{aligned} E_{\alpha }(x) = \sum _{k=0}^{\infty } \frac{x^{k}}{\Gamma (\alpha k + 1)}, \end{aligned}$$

and its general form

9 $$\begin{aligned} E_{\alpha , \beta }(x) = \sum _{k=0}^{\infty } \frac{x^{k}}{\Gamma (\alpha k + \beta )}. \end{aligned}$$

Therefore, (3) in Caputo sense is defined as:

10 $$\begin{aligned} \left\{ \begin{aligned}&^{c}_{0}{\mathcal {D}}^{\alpha }_{t} S(t) = \Lambda ^{\alpha } + \rho ^{\alpha } R - (\eta _{s}^{\alpha } + \omega ^{\alpha } + \mu ^{\alpha })S, \\&^{c}_{0}{\mathcal {D}}^{\alpha }_{t} V(t) = \omega ^{\alpha } S - (\eta _{s}^{\alpha } + \mu ^{\alpha })V, \\&^{c}_{0}{\mathcal {D}}^{\alpha }_{t} E(t) = \eta _{s}^{\alpha }S + (1 - \epsilon ^{\alpha }) \eta _{s}^{\alpha } V - (\tau ^{\alpha } + \mu ^{\alpha })E, \\&^{c}_{0}{\mathcal {D}}^{\alpha }_{t} I_{s}(t) = \sigma ^{\alpha } \tau ^{\alpha } E + \gamma _{1}^{\alpha } I_{a} - (\alpha _{2}^{\alpha } + \delta _{1}^{\alpha } + \mu ^{\alpha })I_{s}, \\&^{c}_{0}{\mathcal {D}}^{\alpha }_{t} I_{a}(t) = \varpi ^{\alpha } \tau ^{\alpha } E - (\gamma _{1}^{\alpha } + \gamma _{2}^{\alpha } + \alpha _{1}^{\alpha } + \mu ^{\alpha })I_{a}, \\&^{c}_{0}{\mathcal {D}}^{\alpha }_{t} I_{m}(t) = (1 - \sigma ^{\alpha } - \varpi ^{\alpha })\tau ^{\alpha } E + \gamma _{2}^{\alpha } I_{a} - (\alpha _{3}^{\alpha } + \delta _{2}^{\alpha } + \mu ^{\alpha })I_{m}, \\&^{c}_{0}{\mathcal {D}}^{\alpha }_{t} R(t) = \epsilon ^{\alpha } \eta _{s}^{\alpha } V + \alpha _{1}^{\alpha } I_{a} + \alpha _{2}^{\alpha } I_{s} + \alpha _{3}^{\alpha } I_{m} - (\rho ^{\alpha } + \mu ^{\alpha }) R, \end{aligned}\right. \end{aligned}$$

where

$$\begin{aligned} \eta _{s}^{\alpha } = \frac{\beta ^{\alpha }(I_{a} + \nu _{1}^{\alpha } I_{s} + \nu _{2}^{\alpha } I_{m})}{N}, \end{aligned}$$

subject to initial conditions

$$\begin{aligned}&S_{0}(t) = S(0) \text {, } V_{0}(t) = V(0) \text {, } E_{0}(t) = E(0) \text {, } I_{s_{0}}(t) = I_{s}(0) \text {, } I_{a_{0}}(t) = I_{a}(0) \text {, } \\&I_{m_{0}}(t) = I_{m}(0) \text {, } R_{0}(t) = R(0). \end{aligned} $$

By incorporating an auxiliary parameter $\varkappa > 0$, we adeptly modify the fractional operator, effectively eliminating any concerns of dimensional mismatching^60^, thus

11 $$\begin{aligned} \left\{ \begin{aligned}&\varkappa ^{\alpha - 1} \text { } ^{c}_{0}{\mathcal {D}}^{\alpha }_{t} S(t) = \Lambda ^{\alpha } + \rho ^{\alpha } R - \big (\eta _{s}^{\alpha } + \omega ^{\alpha } + \mu ^{\alpha } \big )S, \\&\varkappa ^{\alpha - 1} \text { } ^{c}_{0}{\mathcal {D}}^{\alpha }_{t} V(t) = \omega ^{\alpha } S - \big (\eta _{s}^{\alpha } + \mu ^{\alpha } \big )V, \\&\varkappa ^{\alpha - 1} \text { } ^{c}_{0}{\mathcal {D}}^{\alpha }_{t} E(t) = \eta _{s}^{\alpha }S + \big (1 - \epsilon ^{\alpha } \big ) \eta _{s}^{\alpha } V - \big (\tau ^{\alpha } + \mu ^{\alpha } \big )E, \\&\varkappa ^{\alpha - 1} \text { } ^{c}_{0}{\mathcal {D}}^{\alpha }_{t} I_{s}(t) = \sigma ^{\alpha } \tau ^{\alpha } E + \gamma _{1}^{\alpha } I_{a} - \big (\alpha _{2}^{\alpha } + \delta _{1}^{\alpha } + \mu ^{\alpha } \big )I_{s}, \\&\varkappa ^{\alpha - 1} \text { } ^{c}_{0}{\mathcal {D}}^{\alpha }_{t} I_{a}(t) = \varpi ^{\alpha } \tau ^{\alpha } E - \big (\gamma _{1}^{\alpha } + \gamma _{2}^{\alpha } + \alpha _{1}^{\alpha } + \mu ^{\alpha } \big )I_{a}, \\&\varkappa ^{\alpha - 1} \text { } ^{c}_{0}{\mathcal {D}}^{\alpha }_{t} I_{m}(t) = \big (1 - \sigma ^{\alpha } - \varpi ^{\alpha } \big )\tau ^{\alpha } E + \gamma _{2}^{\alpha } I_{a} - \big (\alpha _{3}^{\alpha } + \delta _{2}^{\alpha } + \mu ^{\alpha } \big )I_{m}, \\&\varkappa ^{\alpha - 1} \text { } ^{c}_{0}{\mathcal {D}}^{\alpha }_{t} R(t) = \epsilon ^{\alpha } \eta _{s}^{\alpha } V + \alpha _{1}^{\alpha } I_{a} + \alpha _{2}^{\alpha } I_{s} + \alpha _{3}^{\alpha } I_{m} - \big (\rho ^{\alpha } + \mu ^{\alpha } \big ) R, \end{aligned}\right. \end{aligned}$$

where

$$\begin{aligned} \eta _{s}^{\alpha } = \frac{\beta ^{\alpha } \left( I_{a} + \nu _{1}^{\alpha } I_{s} + \nu _{2}^{\alpha } I_{m} \right) }{N}, \end{aligned}$$

subject to initial conditions

$$\begin{aligned} &S_{0}(t) = S(0) \text {, } V_{0}(t) = V(0) \text {, } E_{0}(t) = E(0) \text {, } I_{s_{0}}(t) = I_{s}(0) \text {, } I_{a_{0}}(t) = I_{a}(0) \text {, } \\&I_{m_{0}}(t) = I_{m}(0) \text {, } R_{0}(t) = R(0). \end{aligned}$$

## Qualitative properties of the model

Analysis of (11) is carried out in this section.

### Boundedness and positivity of the model system (11)

The fractional-order system (11) must be positive because the solutions represent the densities of the populations that interact with each other, and from a biological perspective, the lowest possible value for each population in the model system is zero, which is relevant to establishing an upper bound. This is assured by the following outcomes:

#### Theorem 3

Consider (11) with $S(0)> 0, V(0) > 0, E(0) \ge 0, I_{s}(0) \ge 0, I_{a}(0) \ge 0, I_{m}(0) \ge 0 \text { and } R(0) \ge 0$ as an initial condition, then all solutions are uniformly bounded and positive.

#### Proof

First, we start by adding the population states which is possible by the linearity property of the Caputo fractional derivative:

12 $$\begin{aligned} ^{c}_{0}{\mathcal {D}}^{\alpha }_{t} N(t)&= \, ^{c}_{0}{\mathcal {D}}^{\alpha }_{t} S(t) + ^{c}_{0}{\mathcal {D}}^{\alpha }_{t} V(t) + ^{c}_{0}{\mathcal {D}}^{\alpha }_{t} E(t) + ^{c}_{0}{\mathcal {D}}^{\alpha }_{t} I_{s}(t) + ^{c}_{0}{\mathcal {D}}^{\alpha }_{t} I_{a}(t) + ^{c}_{0}{\mathcal {D}}^{\alpha }_{t} I_{m}(t) + ^{c}_{0}{\mathcal {D}}^{\alpha }_{t} R(t), \\&= \Lambda ^{\alpha } - \delta _{1}^{\alpha } I_{s} - \delta _{2}^{\alpha } I_{m} - \mu ^{\alpha } N, \\&\le \Lambda ^{\alpha } - \mu ^{\alpha } N. \end{aligned}$$

By using Laplace transform and its inverse on (12), after simplifying we obtain,

13 $$\begin{aligned} N(t) \le \frac{\Lambda ^{\alpha }}{\mu ^{\alpha }} \left[ 1 - E_{\alpha ,1} \left( -\mu ^{\alpha } t^{\alpha } \right) \right] + \sum _{k=0}^{n-1} E_{\alpha ,k+1} \left( -\mu ^{\alpha } t^{\alpha } \right) t^{k} N^{(k)}(t_{0}), \end{aligned}$$

where $E_{\alpha ,1}(-\mu ^{\alpha } t^{\alpha }) \text { and } E_{\alpha ,k+1}(-\mu ^{\alpha } t^{\alpha })$ are Mittag-Leffler functions^61^. Hence, solutions of the Caputo (11) confined in the region ${\mathbb {D}}$, where

14 $$\begin{aligned} {\mathbb {D}}&=\left\{ \left( S, V, E, I_{s}, I_{a}, I_{m}, R \right) \in {\mathbb {R}}^{7}: N(t) \le \frac{\Lambda ^{\alpha }}{\mu ^{\alpha }} \left[ 1 - E_{\alpha ,1} \left( -\mu ^{\alpha } t^{\alpha } \right) \right] \right. \\&\quad + \, \left. \sum _{k=0}^{n-1} E_{\alpha ,k+1} \left( -\mu ^{\alpha } t^{\alpha } \right) t^{k} N^{(k)}(t_{0}) \right\} . \end{aligned} $$

Secondly, we show that the solutions of (11) are positive in the feasible region ${\mathbb {D}}$. In order to show this, we begin by examining the first equation of the model (11)

15 $$\begin{aligned} ^{c}_{0}{\mathcal {D}}^{\alpha }_{t} S(t)&= \Lambda ^{\alpha } + \rho ^{\alpha } R - \frac{\beta ^{\alpha } \left( I_{a} + \nu _{1}^{\alpha } I_{s} + \nu _{2}^{\alpha } I_{m} \right) S}{N} - \left( \omega ^{\alpha } + \mu ^{\alpha } \right) S \\&\ge - \frac{\beta ^{\alpha } \left( I_{a} + \nu _{1}^{\alpha } I_{s} + \nu _{2}^{\alpha } I_{m} \right) S}{N} - \left( \omega ^{\alpha } + \mu ^{\alpha } \right) S \\&= - bS, \end{aligned}$$

where $b = \frac{\beta ^{\alpha }(I_{a} + \nu _{1}^{\alpha } I_{s} + \nu _{2}^{\alpha } I_{m})}{N} + (\omega ^{\alpha } + \mu ^{\alpha })$. Using the Laplace transform method and the positivity of the Mittag-Leffler function^62^ we have

$$\begin{aligned} S(t) \ge S(0) \sum _{k=0}^{n-1} E_{\alpha , k+1}(-bt^{\alpha })t^{k} \Longrightarrow S \ge 0. \end{aligned}$$

In a similar manner, $V(t), E(t), I_{s}, I_{a}, I_{m}, R \ge 0, \text { } \forall t \ge 0$. 

### Existence and uniqueness of solutions of (11)

Consider a Banach space on $J = [0, T]$ of all continuous real-valued functions denoted as ${\mathcal {B}}(J, {\mathbb {R}})$ with the following norm:

$$\begin{aligned} ||(S, V, E, I_{s}, I_{a}, I_{m}, R)|| = ||S(t)||+||V(t)||+||E(t)||+||I_{s}(t)||+||I_{a}(t)||+||I_{m}(t)||+||R(t)||, \end{aligned}$$

such as

$$\begin{aligned}{} & {} ||S(t)|| = \sup _{t \in [0, T]}|S(t)|, ||V(t)|| = \sup _{t \in [0, T]}|V(t)|, ||E(t)|| = \sup _{t \in [0, T]}|E(t)|, ||I_{s}(t)|| = \sup _{t \in [0, T]}|I_{s}(t)|,\\{} & {} ||I_{a}(t)|| = \sup _{t \in [0, T]}|I_{a}(t)|, ||I_{m}(t)|| = \sup _{t \in [0, T]}|I{m}(t)| \text { and } ||R(t)|| = \sup _{t \in [0, T]}|R(t)|. \end{aligned}$$

Applying (7) on both sides of (11) we obtain:

16 $$\begin{aligned} S(t) - S(0)&= ^{c}{\mathcal {I}}^{\alpha }_{t} \big \{ \Lambda ^{\alpha } + \rho ^{\alpha } R - \big (\eta _{s}^{\alpha } + \omega ^{\alpha } + \mu ^{\alpha } \big )S \big \}, \\ V(t) - V(0)&= ^{c}{\mathcal {I}}^{\alpha }_{t} \big \{ \omega ^{\alpha } S - (\eta _{s}^{\alpha } + \mu ^{\alpha })V \big \}, \\ E(t) - E(0)&= ^{c}{\mathcal {I}}^{\alpha }_{t} \{ \eta _{s}^{\alpha } \big (S + \big (1 - \epsilon ^{\alpha } \big )V \big ) - \big (\tau ^{\alpha } + \mu ^{\alpha } \big )E \big \}, \\ I_{s}(t) - I_{s}(0)&= ^{c}{\mathcal {I}}^{\alpha }_{t} \big \{ \sigma ^{\alpha } \tau ^{\alpha } E + \gamma _{1}^{\alpha } I_{a} - \big (\alpha _{2}^{\alpha } + \delta _{1}^{\alpha } + \mu ^{\alpha } \big )I_{s} \big \}, \\ I_{a}(t) - I_{a}(0)&= ^{c}{\mathcal {I}}^{\alpha }_{t} \big \{ \varpi ^{\alpha } \tau ^{\alpha } E - \big (\gamma _{1}^{\alpha } + \gamma _{2}^{\alpha } + \alpha _{1}^{\alpha } + \mu ^{\alpha }\big )I_{a} \big \}, \\ I_{m}(t) - I_{m}(0)&= ^{c}{\mathcal {I}}^{\alpha }_{t} \big \{ \big (1 - \sigma ^{\alpha } - \varpi ^{\alpha } \big )\tau ^{\alpha } E + \gamma _{2}^{\alpha } I_{a} - \big (\alpha _{3}^{\alpha } + \delta _{2}^{\alpha } + \mu ^{\alpha } \big )I_{m} \big \}, \\ R(t) - R(0)&= ^{c}{\mathcal {I}}^{\alpha }_{t} \big \{ \epsilon ^{\alpha } \eta _{s}^{\alpha } + \alpha _{1}^{\alpha } I_{a} + \alpha _{2}^{\alpha } I_{s} + \alpha _{3}^{\alpha } I_{m} - \big (\rho ^{\alpha } + \mu ^{\alpha } \big ) R \big \}, \end{aligned}$$

The definition (7) then directs us to the following:

17 $$\begin{aligned} S(t)&= S(0) + \frac{1}{\Gamma (\alpha )} \int _{0}^{t} (t - \xi )^{ \alpha - 1} G_{1}(\xi , S(\xi )) d \xi , \\ V(t)&= V(0) + \frac{1}{\Gamma (\alpha )} \int _{0}^{t} (t - \xi )^{ \alpha - 1} G_{2}(\xi , V(\xi )) d \xi , \\ E(t)&= E(0) + \frac{1}{\Gamma (\alpha )} \int _{0}^{t} (t - \xi )^{ \alpha - 1} G_{3}(\xi , E(\xi )) d \xi , \\ I_{s}(t)&= I_{s}(0) + \frac{1}{\Gamma (\alpha )} \int _{0}^{t} (t - \xi )^{ \alpha - 1} G_{4}(\xi , I_{s}(\xi )) d \xi , \\ I_{a}(t)&= I_{a}(0) + \frac{1}{\Gamma (\alpha )} \int _{0}^{t} (t - \xi )^{ \alpha - 1} G_{5}(\xi , I_{a}(\xi )) d \xi , \\ I_{m}(t)&= I_{m}(0) + \frac{1}{\Gamma (\alpha )} \int _{0}^{t} (t - \xi )^{ \alpha - 1} G_{6}(\xi , I_{m}(\xi )) d \xi , \\ R(t)&= R(0) + \frac{1}{\Gamma (\alpha )} \int _{0}^{t} (t - \xi )^{ \alpha - 1} G_{7}(\xi , R(\xi )) d \xi . \end{aligned}$$

with the respective kernels

18 $$\begin{aligned} G_{1}(t, S(t))&= \Lambda ^{\alpha } + \rho ^{\alpha } R - \big (\eta _{s}^{\alpha } + \omega ^{\alpha } + \mu ^{\alpha } \big )S, \\ G_{2}(t, V(t))&= \omega ^{\alpha } S - \big (\eta _{s}^{\alpha } + \mu ^{\alpha } \big )V, \\ G_{3}(t, E(t))&= \eta _{s}^{\alpha } \big (S + \big (1 - \epsilon ^{\alpha } \big )V \big ) - \big (\tau ^{\alpha } + \mu ^{\alpha } \big )E, \\ G_{4} \big (t, I_{s}(t) \big )&= \sigma ^{\alpha } \tau ^{\alpha } E + \gamma _{1}^{\alpha } I_{a} - \big (\alpha _{2}^{\alpha } + \delta _{1}^{\alpha } + \mu ^{\alpha } \big )I_{s}, \\ G_{5}\big (t, I_{a}(t) \big )&= \varpi ^{\alpha } \tau ^{\alpha } E - \big (\gamma _{1}^{\alpha } + \gamma _{2}^{\alpha } + \alpha _{1}^{\alpha } + \mu ^{\alpha } \big )I_{a}, \\ G_{6}\big (t, I_{m}(t) \big )&= \big (1 - \sigma ^{\alpha } - \varpi ^{\alpha } \big )\tau ^{\alpha } E + \gamma _{2}^{\alpha } I_{a} - \big (\alpha _{3}^{\alpha } + \delta _{2}^{\alpha } + \mu ^{\alpha } \big )I_{m}, \\ G_{7}(t, R(t))&= \epsilon ^{\alpha } \eta _{s}^{\alpha } + \alpha _{1}^{\alpha } I_{a} + \alpha _{2}^{\alpha } I_{s} + \alpha _{3}^{\alpha } I_{m} - \big (\rho ^{\alpha } + \mu ^{\alpha } \big ) R. \end{aligned}$$

An upper bound on $S(t), V(t), E(t), I_{s}(t), I_{a}(t), I_{m}(t), \text { and } R(t)$ is needed for the Lipschitz condition to be satisfied by the kernels $(G_{i}, i=1, 2, \ldots , 7)$ in (18). Consider two distinct function $S \text { and } {\overline{S}}$, then

19 $$\begin{aligned} \left| \left| G_{1}(t, S(t)) - G_{1} \left( t, {\overline{S}}(t) \right) \right| \right| = \left| \left| - \left( \eta _{s}^{\alpha } + \omega ^{\alpha } + \mu ^{\alpha } \right) \left( S(t) - {\overline{S}}(t) \right) \right| \right| . \end{aligned}$$

If we consider

$$\begin{aligned} \zeta _{1} = \left| \left| - \left( \eta _{s}^{\alpha } + \omega ^{\alpha } + \mu ^{\alpha } \right) \right| \right| \end{aligned}$$

we have

$$\begin{aligned} \left| \left| G_{1} (t, S(t)) - G_{1} \left( t, {\overline{S}}(t) \right) \right| \right| \le \zeta _{1} \left| \left| S(t) - {\overline{S}}(t) \right| \right| . \end{aligned}$$

In a similar manner, we also have that for the remaining state variables

20 $$\begin{aligned} \big |\big |G_{2}(t, V(t)) - G_{2}\big (t, {\overline{V}}(t)\big )\big |\big |&\le \zeta _{2}\big |\big |V(t) - {\overline{V}}(t)\big |\big |, \\ \big |\big |G_{3}(t, E(t)) - G_{3}\big (t, {\overline{E}}(t)\big )\big |\big |&\le \zeta _{3} \big |\big |E(t) - {\overline{E}}(t) \big |\big |, \\ \big |\big |G_{4} \big (t, I_{s}(t) \big ) - G_{4} \big (t, \overline{I_{s}}(t) \big )\big |\big |&\le \zeta _{4} \big |\big |I_{s}(t) - \overline{I_{s}}(t) \big |\big |, \\ \big |\big |G_{5} \big (t, I_{a}(t) \big ) - G_{5} \big (t, \overline{I_{a}}(t)\big ) \big |\big |&\le \zeta _{5} \big |\big | I_{a}(t) - \overline{I_{a}}(t) \big |\big |, \\ \big |\big |G_{6} \big (t, I_{m}(t) \big ) - G_{6} \big (t, \overline{I_{m}}(t) \big ) \big |\big |&\le \zeta _{6} \big |\big |I_{m}(t) - \overline{I_{m}}(t) \big |\big |, \\ \big |\big |G_{7}(t, R(t)) - G_{7}\big (t, {\overline{R}}(t) \big ) \big |\big |&\le \zeta _{7} \big |\big |R(t) - {\overline{R}}(t) \big |\big |, \end{aligned}$$

where

21 $$\begin{aligned} \zeta _{2}&= \big |\big |- \big (\eta _{s}^{\alpha } + \mu ^{\alpha } \big ) \big |\big |, \\ \zeta _{3}&= \big |\big |-\big (\tau ^{\alpha } + \mu ^{\alpha }\big )\big |\big |, \\ \zeta _{4}&= \big |\big |-\big (\alpha _{2}^{\alpha } + \delta _{1}^{\alpha } + \mu ^{\alpha }\big )\big |\big |, \\ \zeta _{5}&= \big |\big |-\big (\gamma _{1}^{\alpha } + \gamma _{2}^{\alpha } + \alpha _{1}^{\alpha } + \mu ^{\alpha }\big )\big |\big |, \\ \zeta _{6}&= \big |\big |-\big (\alpha _{3}^{\alpha } + \delta _{2}^{\alpha } + \mu ^{\alpha }\big )\big |\big |, \\ \zeta _{7}&= \big |\big |-\big (\rho ^{\alpha } + \mu ^{\alpha }\big )\big |\big |. \end{aligned}$$

The Lipschitz constants for each kernel $G_{i}, i=1,2, \ldots ,7$ are asserted by $\zeta _{1}, \zeta _{2}, \ldots , \zeta _{7}$ respectively. As a result, the Lipschitz condition is satisfied.

Using (17) the following recursive formulae can now be used in order to establish the uniqueness:

22 $$\begin{aligned} S_{n}(t)&= S(0) + \frac{1}{\Gamma (\alpha )} \int _{0}^{t} (t - \xi )^{ \alpha - 1} G_{1}(\xi , S_{n-1}(\xi )) d \xi , \\ V_{n}(t)&= V(0) + \frac{1}{\Gamma (\alpha )} \int _{0}^{t} (t - \xi )^{ \alpha - 1} G_{2}(\xi , V_{n-1}(\xi )) d \xi , \\ E_{n}(t)&= E(0) + \frac{1}{\Gamma (\alpha )} \int _{0}^{t} (t - \xi )^{ \alpha - 1} G_{3}(\xi , E_{n-1}(\xi )) d \xi , \\ I_{s_{n}}(t)&= I_{s}(0) + \frac{1}{\Gamma (\alpha )} \int _{0}^{t} (t - \xi )^{ \alpha - 1} G_{4}(\xi , I_{s_{(n-1)}}(\xi )) d \xi , \\ I_{a_{n}}(t)&= I_{a}(0) + \frac{1}{\Gamma (\alpha )} \int _{0}^{t} (t - \xi )^{ \alpha - 1} G_{5}(\xi , I_{a_{(n-1)}}(\xi )) d \xi , \\ I_{m_{n}}(t)&= I_{m}(0) + \frac{1}{\Gamma (\alpha )} \int _{0}^{t} (t - \xi )^{ \alpha - 1} G_{6}(\xi , I_{m_{(n-1)}}(\xi )) d \xi , \\ R_{n}(t)&= R(0) + \frac{1}{\Gamma (\alpha )} \int _{0}^{t} (t - \xi )^{ \alpha - 1} G_{7}(\xi , R_{n-1}(\xi )) d \xi . \end{aligned}$$

In recursive formulas, the difference between the consecutive terms can be written as follows:

23 $$\begin{aligned} \Upsilon _{n}^{1}(t)&= S_{n}(t) - S_{n-1}(t) = \frac{1}{\Gamma (\alpha )} \int _{0}^{t} (t - \xi )^{ \alpha - 1} (G_{1}(\xi , S_{n-1}(\xi )) - G_{1}(\xi , S_{n-2}(\xi ))) d \xi , \\ \Upsilon _{n}^{2}(t)&= V_{n}(t) - V_{n-1}(t) = \frac{1}{\Gamma (\alpha )} \int _{0}^{t} (t - \xi )^{ \alpha - 1} (G_{2}(\xi , V_{n-1}(\xi )) - G_{2}(\xi , V_{n-2}(\xi ))) d \xi , \\ \Upsilon _{n}^{3}(t)&= E_{n}(t) - E_{n-1}(t) = \frac{1}{\Gamma (\alpha )} \int _{0}^{t} (t - \xi )^{ \alpha - 1} (G_{3}(\xi , E_{n-1}(\xi )) - G_{3}(\xi , E_{n-2}(\xi ))) d \xi , \\ \Upsilon _{n}^{4}(t)&= I_{s_{n}}(t) - I_{s_{n-1}}(t) = \frac{1}{\Gamma (\alpha )} \int _{0}^{t} (t - \xi )^{ \alpha - 1} (G_{4}(\xi , I_{s_{(n-1)}}(\xi )) - G_{4}(\xi , I_{s_{(n-2)}}(\xi ))) d \xi , \\ \Upsilon _{n}^{5}(t)&= I_{a_{n}}(t) - I_{a_{n-1}}(t) = \frac{1}{\Gamma (\alpha )} \int _{0}^{t} (t - \xi )^{ \alpha - 1} (G_{5}(\xi , I_{a_{(n-1)}}(\xi )) - G_{5}(\xi , I_{a_{(n-2)}}(\xi ))) d \xi , \\ \Upsilon _{n}^{6}(t)&= I_{m_{n}}(t) - I_{m_{n-1}}(t) = \frac{1}{\Gamma (\alpha )} \int _{0}^{t} (t - \xi )^{ \alpha - 1} (G_{6}(\xi , I_{m_{(n-1)}}(\xi )) - G_{6}(\xi , I_{m_{(n-2)}}(\xi ))) d \xi , \\ \Upsilon _{n}^{7}(t)&= R_{n}(t) - R_{n-1}(t) = \frac{1}{\Gamma (\alpha )} \int _{0}^{t} (t - \xi )^{ \alpha - 1} (G_{7}(\xi , R_{n-1}(\xi )) - G_{7}(\xi , R_{n-2}(\xi ))) d \xi . \end{aligned}$$

Note that

$$\begin{aligned}{} & {} S_{n}(t) = \sum _{i=0}^{n} \Upsilon _{i}^{1}(t), V_{n}(t) = \sum _{i=0}^{n} \Upsilon _{i}^{2}(t), E_{n}(t) = \sum _{i=0}^{n} \Upsilon _{i}^{3}(t), I_{s_{n}}(t) = \sum _{i=0}^{n} \Upsilon _{i}^{4}(t), \\{} & {} I_{a_{n}}(t) = \sum _{i=0}^{n} \Upsilon _{i}^{5}(t), I_{m_{n}}(t) = \sum _{i=0}^{n} \Upsilon _{i}^{6}(t), R_{n}(t) = \sum _{i=0}^{n} \Upsilon _{i}^{7}(t). \end{aligned}$$

Applying the norm, for each of the differences in (23) we formulate the recursive inequalities as follows:

24 $$\begin{aligned} ||\Upsilon _{n}^{1}(t)||&= ||S_{n}(t) - S_{n-1}(t)|| \\&= \frac{1}{\Gamma (\alpha )} \int _{0}^{t} (t - \xi )^{ \alpha - 1} ||(G_{1}(\xi , S_{n-1}(\xi )) - G_{1}(\xi , S_{n-2}(\xi )))|| d \xi . \end{aligned}$$

Since the kernel $G_{1}$ satisfies the Lipschitz condition with constant $\zeta _{1}$, then we can see that

$$\begin{aligned} ||S_{n}(t) - S_{n-1}(t)|| = \frac{1}{\Gamma (\alpha )} \int _{0}^{t} (t - \xi )^{ \alpha - 1} \zeta _{1} ||S_{n-1}(\xi ) - S_{n-2}(\xi )|| d \xi . \end{aligned}$$

Thus, we obtain

25 $$\begin{aligned} ||\Upsilon _{n}^{1}(t)|| = \frac{\zeta _{1}}{\Gamma (\alpha )} \int _{0}^{t} (t - \xi )^{ \alpha - 1} ||\Upsilon _{n-1}^{1}(\xi )|| d \xi . \end{aligned}$$

As a result, we can obtain the following:

26 $$\begin{aligned} \big |\big |\Upsilon _{n}^{2}(t)\big |\big |&= \frac{\zeta _{2}}{\Gamma (\alpha )} \int _{0}^{t} (t - \xi )^{ \alpha - 1} \big |\big |\Upsilon _{n-1}^{2}(\xi ) \big |\big | d \xi . \\ \big |\big |\Upsilon _{n}^{3}(t) \big |\big |&= \frac{\zeta _{3}}{\Gamma (\alpha )} \int _{0}^{t} (t - \xi )^{ \alpha - 1} \big |\big |\Upsilon _{n-1}^{3}(\xi ) \big |\big | d \xi . \\ \big |\big |\Upsilon _{n}^{4}(t) \big |\big |&= \frac{\zeta _{4}}{\Gamma (\alpha )} \int _{0}^{t} (t - \xi )^{ \alpha - 1} \big |\big |\Upsilon _{n-1}^{4}(\xi ) \big |\big | d \xi . \\ \big |\big |\Upsilon _{n}^{5}(t) \big |\big |&= \frac{\zeta _{5}}{\Gamma (\alpha )} \int _{0}^{t} (t - \xi )^{ \alpha - 1} \big |\big |\Upsilon _{n-1}^{5}(\xi ) \big |\big | d \xi . \\ \big |\big |\Upsilon _{n}^{6}(t) \big |\big |&= \frac{\zeta _{6}}{\Gamma (\alpha )} \int _{0}^{t} (t - \xi )^{ \alpha - 1} \big |\big |\Upsilon _{n-1}^{6}(\xi ) \big |\big | d \xi . \\ \big |\big |\Upsilon _{n}^{7}(t) \big |\big |&= \frac{\zeta _{7}}{\Gamma (\alpha )} \int _{0}^{t} (t - \xi )^{ \alpha - 1} \big |\big |\Upsilon _{n-1}^{7}(\xi ) \big |\big | d \xi . \end{aligned}$$

#### Theorem 4

Suppose for $t \in [0, b]$ the following inequalities hold:

$$\begin{aligned} \frac{\zeta _{i} b^{\alpha }}{\Gamma (\alpha )} < 1, i = 1,2, \ldots ,7. \end{aligned}$$

Then the model (11) has a unique solution

#### Proof

Recursively solving Eqs. (25)–(26) yields the following relations when the functions $S(t), V(t), E(t), I_{s}(t), I_{a}(t), I_{m}(t) \text {, and } R(t)$ are assumed to be bounded and each kernel satisfies a Lipschitz condition:

27 $$\begin{aligned} ||\Upsilon _{n}^{1}(t)||&\le ||S_{0}(t)||\left[ \frac{\zeta _{1} b^{\alpha }}{\Gamma (\alpha )}\right] ^{n}, \\ ||\Upsilon _{n}^{2}(t)||&\le ||V_{0}(t)||\left[ \frac{\zeta _{2} b^{\alpha }}{\Gamma (\alpha )}\right] ^{n}, \\ ||\Upsilon _{n}^{3}(t)||&\le ||E_{0}(t)||\left[ \frac{\zeta _{3} b^{\alpha }}{\Gamma (\alpha )}\right] ^{n}, \\ ||\Upsilon _{n}^{4}(t)||&\le ||I_{s_{0}}(t)||\left[ \frac{\zeta _{4} b^{\alpha }}{\Gamma (\alpha )}\right] ^{n}, \\ ||\Upsilon _{n}^{5}(t)||&\le ||I_{a_{0}}(t)||\left[ \frac{\zeta _{5} b^{\alpha }}{\Gamma (\alpha )}\right] ^{n}, \\ ||\Upsilon _{n}^{6}(t)||&\le ||I_{m_{0}}(t)||\left[ \frac{\zeta _{6} b^{\alpha }}{\Gamma (\alpha )}\right] ^{n}, \\ ||\Upsilon _{n}^{7}(t)||&\le ||R_{0}(t)||\left[ \frac{\zeta _{7} b^{\alpha }}{\Gamma (\alpha )}\right] ^{n}. \end{aligned}$$

Thus, it can be observed that the sequence (27) satisfy $||\Upsilon _{n}^{i}(t)|| \Longrightarrow 0 \text { , for } i=1, 2, \ldots ,7 \text { as } n \Longrightarrow \infty$. Further, by applying the triangular inequality to equation (27) and for any *k*, we are able to find:

28 $$\begin{aligned} ||S_{n+k}(t) - S_{n}(t)||&\le \sum _{j=n+1}^{n+k} q_{1}^{j} = \frac{q_{1}^{n+1} + q_{1}^{n+k+1}}{1 - q_{1}}, \\ ||V_{n+k}(t) - V_{n}(t)||&\le \sum _{j=n+1}^{n+k} q_{2}^{j} = \frac{q_{2}^{n+1} + q_{2}^{n+k+1}}{1 - q_{2}}, \\ ||E_{n+k}(t) - E_{n}(t)||&\le \sum _{j=n+1}^{n+k} q_{3}^{j} = \frac{q_{3}^{n+1} + q_{3}^{n+k+1}}{1 - q_{3}}, \\ ||I_{s_{n+k}}(t) - I_{s_{n}}(t)||&\le \sum _{j=n+1}^{n+k} q_{4}^{j} = \frac{q_{4}^{n+1} + q_{4}^{n+k+1}}{1 - q_{4}}, \\ ||I_{a_{n+k}}(t) - I_{a_{n}}(t)||&\le \sum _{j=n+1}^{n+k} q_{5}^{j} = \frac{q_{5}^{n+1} + q_{5}^{n+k+1}}{1 - q_{5}}, \\ ||I_{m_{n+k}}(t) - I_{m_{n+k}}(t)||&\le \sum _{j=n+1}^{n+k} q_{6}^{j} = \frac{q_{6}^{n+1} + q_{6}^{n+k+1}}{1 - q_{6}}, \\ ||R_{n+k}(t) - R_{n}(t)||&\le \sum _{j=n+1}^{n+k} q_{7}^{j} = \frac{q_{7}^{n+1} + q_{7}^{n+k+1}}{1 - q_{7}}, \end{aligned}$$

where $q_{i}'s$ are by hypothesis $q_{i}'s = \frac{\zeta _{i} b^{\alpha }}{\Gamma (\alpha )} < 1 \text { ,for } i = 1, 2, \ldots 7$. Thus, by (27), a Cauchy sequence in ${\mathcal {B}}$ is formed by $S_{n}, V_{n}, E_{n}, I_{s_{n}}, I_{a_{n}}, I_{m_{n}}, \text { and } R_{n}$. Hence as $n \Longrightarrow \infty$, the unique solution of (11) is obtained.

### Equilibrium point and stability analysis

In this section, we find the disease-free equilibrium point (DFE) and the endemic equilibria (EE). The next-generation matrix is used to calculate the effective reproduction number $R_{eff}$. Lyapunov functions were constructed and used to establish the global stability of the equilibria.

#### Disease free equilibrium (DFE)

First setting (11) to 0 we get the following distinct DFE solutions

29 $$\begin{aligned} DFE = \left( S^{0}, V^{0}, E^{0}, I_{s}^{0}, I_{a}^{0}, I_{m}^{0}, R^{0} \right) = \left( \frac{\Lambda ^{\alpha }}{\omega ^{\alpha } + \mu ^{\alpha }}, \frac{\omega ^{\alpha } \Lambda ^{\alpha }}{\mu ^{\alpha }(\omega ^{\alpha } + \mu ^{\alpha })}, 0, 0, 0, 0, 0\right) . \end{aligned}$$

#### Effective reproduction number ($R_{eff}$)

We use the method in^63^ similarly to^64,65^ to compute $R_{eff}$. Let *F* represent non-negative matrix of the new infection, *V* is the transmission matrix, then:

30 $$\begin{aligned} F = \begin{pmatrix} 0 &{} \frac{k_{1} \beta ^{\alpha } \nu _{1}^{\alpha }}{k_{2}} &{} \frac{k_{1} \beta ^{\alpha }}{k_{2}} &{} \frac{k_{1} \beta ^{\alpha } \nu _{2}^{\alpha }}{k_{2}} \\ 0 &{}\quad 0 &{}\quad 0 &{}\quad 0 \\ 0 &{}\quad 0 &{}\quad 0 &{}\quad 0 \\ 0 &{}\quad 0 &{}\quad 0 &{}\quad 0 \end{pmatrix}, V = \begin{pmatrix} k_{3} &{}\quad 0 &{}\quad 0 &{}\quad 0 \\ - \sigma ^{\alpha } \tau ^{\alpha } &{}\quad k_{4} &{}\quad -\gamma _{1}^{\alpha } &{}\quad 0\\ -\varphi ^{\alpha } \tau ^{\alpha } &{}\quad 0 &{}\quad k_{5} &{}\quad 0 \\ -k_{6} \tau ^{\alpha } &{}\quad 0 &{}\quad -\gamma _{2}^{\alpha } &{}\quad k_{7} \end{pmatrix}. \end{aligned}$$

where

$$\begin{aligned}{} & {} k_{1} = (1-\epsilon ^{\alpha })\omega ^{\alpha } + \mu ^{\alpha } \text {, } k_{2} = \omega ^{\alpha } + \mu ^{\alpha } \text {, } k_{3} = \tau ^{\alpha } + \mu ^{\alpha } \text {, } k_{4} = \alpha _{2}^{\alpha } + \delta _{1}^{\alpha } + \mu ^{\alpha } \text {, } k_{5} = \gamma _{1}^{\alpha } + \gamma _{2}^{\alpha } + \alpha _{1}^{\alpha } + \mu ^{\alpha } \text {, } \\{} & {} k_{6}= 1 - \sigma ^{\alpha } - \varpi ^{\alpha } \text {, } k_{7} = \alpha _{3}^{\alpha } + \delta _{2}^{\alpha } + \mu ^{\alpha } \text {, and} k_{8} = \rho ^{\alpha } + \mu ^{\alpha }. \end{aligned}$$

Therefore,

31 $$\begin{aligned} R_{eff} = \frac{k_{1} \beta ^{\alpha } \tau ^{\alpha }((\varpi ^{\alpha } k_{7} + \nu _{2}^{\alpha }(\varpi ^{\alpha } \gamma _{2}^{\alpha } + k_{5} k_{6}))k_{4}+ k_{7} \nu _{1}^{\alpha }(\sigma ^{\alpha } k_{5} + \varpi ^{\alpha } \gamma _{1}^{\alpha }))}{k_{2}k_{3}k_{4}k_{5}k_{7}}. \end{aligned}$$

Thus, $R_{eff}$ can be expressed as:

32 $$\begin{aligned} R_{eff} = R_{ea} + R_{es} + R_{em} \end{aligned}$$

where

$$\begin{aligned} R_{ea} = \frac{k_{1} \beta ^{\alpha } \tau ^{\alpha } \varpi ^{\alpha }}{k_{2}k_{3}k_{5}} \text {, } R_{es} = \frac{k_{1} \beta ^{\alpha } \tau ^{\alpha } (\varpi ^{\alpha } \gamma _{1}^{\alpha } + \sigma ^{\alpha } k_{5})\nu _{1}^{\alpha }}{k_{2}k_{3}k_{4}k_{5}} \text {, and } R_{em} = \frac{k_{1} \beta ^{\alpha } \tau ^{\alpha } (\varpi ^{\alpha } \gamma _{2}^{\alpha } + k_{5}k_{6})\nu _{2}}{k_{2}k_{3}k_{5}k_{7}}. \end{aligned}$$

Hence, we have the following lemma:

##### Lemma 5

The DFE is locally asymptotically stable if $R_{eff} < 1$, and unstable otherwise.

For global stability, consider the following theorem:

##### Theorem 6

The DFE is globally asymptotically stable if $R_{eff} \le 1$.

##### Proof

Consider the Lyapunov function:

33 $$\begin{aligned} F_{1} = L_{1}E + L_{2}I_{s} + L_{3}I_{a} + L_{4}I_{m}, \end{aligned}$$

where $$L_{1} = \frac{\tau ^{\alpha } (\nu _{1}^{\alpha } k_{7}(\varpi ^{\alpha } \gamma _{1}^{\alpha } + \sigma ^{\alpha } k_{5}) + k_{4}(\varpi ^{\alpha } k_{7} + \nu _{2}^{\alpha } (\varpi ^{\alpha } \gamma _{2}^{\alpha } + k_{5}k_{6})))}{k_{3} k_{4} k_{5} k_{7}}, L_{2} = \frac{\nu _{1}^{\alpha }}{k_{4}}, L_{3} = \frac{k_{4} \gamma _{2}^{\alpha } \nu _{2}^{\alpha } + k_{7} \gamma _{1}^{\alpha } \nu _{1}^{\alpha } + k_{4}k_{7}}{k_{4}k_{5}k_{7}},$$ $$L_{4} = \frac{\nu _{2}^{\alpha }}{k_{7}}.$$

The Lyapunov derivative is calculated as

$$\begin{aligned} \dot{F_{1}}&= \frac{\tau ^{\alpha } \left( \nu _{1}^{\alpha } k_{7} \left( \varpi ^{\alpha } \gamma _{1}^{\alpha } + \sigma ^{\alpha } k_{5} \right) + k_{4} \left( \varpi ^{\alpha } k_{7} + \nu _{2}^{\alpha } \left( \varpi ^{\alpha } \gamma _{2}^{\alpha } + k_{5}k_{6} \right) \right) \right) }{k_{3} k_{4} k_{5} k_{7}} \left( \eta _{s}^{\alpha }S + \left( 1 - \epsilon ^{\alpha } \right) \eta _{s}^{\alpha }V - k_{3} E \right) \\&\quad + \frac{\nu _{1}^{\alpha }}{k_{4}} \left( \sigma ^{\alpha } \tau ^{\alpha } E + \gamma _{1}^{\alpha } I_{a} - k_{4} I_{s} \right) + \frac{k_{4} \gamma _{2}^{\alpha } \nu _{2}^{\alpha } + k_{7} \gamma _{1}^{\alpha }\nu _{1}^{\alpha } + k_{4}k_{7}}{k_{4}k_{5}k_{7}} \left( \varpi ^{\alpha } \tau ^{\alpha } E - k_{5} I_{a} \right) \\&\quad + \frac{\nu _{2}^{\alpha }}{k_{7}} \left( k_{6} \tau ^{\alpha } E + \gamma _{2}^{\alpha } I_{a} - k_{7} I_{m} \right) \\&= \frac{\tau ^{\alpha } \nu _{1}^{\alpha } \varpi ^{\alpha } \gamma _{1}^{\alpha } \eta _{s}^{\alpha } S}{k_{3}k_{4}k_{5}} + \frac{\tau ^{\alpha } \nu _{2}^{\alpha } \varpi ^{\alpha } \gamma _{2}^{\alpha } \eta _{s}^{\alpha } S}{k_{3}k_{5}k_{7}} + \frac{\tau ^{\alpha } \nu _{1}^{\alpha } \sigma ^{\alpha } \eta _{s}^{\alpha } S}{k_{3}k_{4}} + \frac{\tau ^{\alpha } \nu _{2}^{\alpha } k_{5} \eta _{s}^{\alpha } S}{k_{3}k_{7}} + \frac{\tau ^{\alpha } \varphi ^{\alpha } \eta _{s}^{\alpha } S}{k_{3}k_{5}} \\&\quad + \frac{\tau ^{\alpha } \nu _{1}^{\alpha } \varpi ^{\alpha } \gamma _{1}^{\alpha } \eta _{s}^{\alpha } \left( 1 - \epsilon ^{\alpha } \right) V}{k_{3}k_{4}k_{5}} + \frac{\tau ^{\alpha } \nu _{2}^{\alpha } \varpi ^{\alpha } \gamma _{2}^{\alpha } \eta _{s}^{\alpha } \left( 1 - \epsilon ^{\alpha } \right) V}{k_{3}k_{5}k_{7}} + \frac{\tau ^{\alpha } \nu _{1}^{\alpha } \sigma ^{\alpha } \eta _{s}^{\alpha } \left( 1 - \epsilon ^{\alpha } \right) V}{k_{3}k_{4}} \\&\quad + \frac{\tau ^{\alpha } \nu _{2}^{\alpha } k_{5} \eta _{s}^{\alpha } \left( 1 - \epsilon ^{\alpha } \right) V}{k_{3}k_{7}} + \frac{\tau ^{\alpha } \varpi ^{\alpha } \eta _{s}^{\alpha } \left( 1 - \epsilon ^{\alpha } \right) V}{k_{3}k_{5}} - I_{a} - \nu _{1}^{\alpha }I_{s} - \nu _{2}^{\alpha } I_{m} \\&= \frac{ \left( \nu _{1}^{\alpha } k_{7} \left( \sigma ^{\alpha } k_{5} + \varpi ^{\alpha } \gamma _{1}^{\alpha } \right) + k_{4} \left( \varpi ^{\alpha } k_{7} + \nu _{2}^{\alpha } \left( \varpi ^{\alpha } \gamma _{2}^{\alpha } + k_{5} k_{6} \right) \right) \right) \tau ^{\alpha } \left( S + \left( 1 - \epsilon ^{\alpha } \right) V \right) \eta _{s}^{\alpha }}{k_{3}k_{4}k_{5}k_{7}} - \left( I_{a} + \nu _{1}^{\alpha }I_{s} + \nu _{2}^{\alpha } I_{m} \right) \\&\le \frac{k_{1} \beta ^{\alpha } \tau ^{\alpha } \left( \nu _{1}^{\alpha } k_{7} \left( \sigma ^{\alpha } k_{5} + \varpi ^{\alpha } \gamma _{1}^{\alpha } \right) + k_{4} \left( \varpi ^{\alpha } k_{7} + \nu _{2}^{\alpha } \left( \varpi ^{\alpha } \gamma _{2}^{\alpha } + k_{5} k_{6} \right) \right) \right) \left( I_{a} + \nu _{1}^{\alpha }I_{s} + \nu _{2}^{\alpha } I_{m} \right) }{k_{2}k_{3}k_{4}k_{5}k_{7}} \\&\quad - \left( I_{a} + \nu _{1}^{\alpha }I_{s} + \nu _{2}^{\alpha } I_{m} \right) . \end{aligned}$$

Hence

34 $$\begin{aligned} \dot{F_{1}} \le \left( I_{a} + \nu _{1}^{\alpha }I_{s} + \nu _{2}^{\alpha }I_{m} \right) (R_{eff} - 1). \end{aligned}$$

#### Existence of endemic equilibrium point (EE) and Bifurcation Analysis

The endemic equilibrium point (EE) represents the situation in which the disease continue to exist across the population.

35 $$\begin{aligned} EE = (S^*, V^*, E^*, I_{s}^*, I_{a}^*, I_{m}^*, R) \end{aligned}$$

36 $$ \begin{aligned}&S^* = \frac{((\eta _{s}^{\alpha })^* + \mu )(\Phi \tau ^{\alpha } \rho ^{\alpha } E^{*} + \Lambda ^{\alpha } k_{4}k_{5}k_{7}k_{8})}{k_{4}k_{5}k_{7}(((\eta _{s}^{\alpha })^* + k_{2})((\eta _{s}^{\alpha })^* + \mu ^{\alpha })k_{8} - \epsilon ^{\alpha } \omega ^{\alpha } \rho ^{\alpha } (\eta _{s}^{\alpha })^*)}, \\&V^* = \frac{\omega ^{\alpha }(\Phi \tau ^{\alpha } \rho ^{\alpha } E^{*} + \Lambda ^{\alpha } k_{4}k_{5}k_{7}k_{8})}{k_{4}k_{5}k_{7}(((\eta _{s}^{\alpha })^* + k_{2})((\eta _{s}^{\alpha })^* + \mu ^{\alpha })k_{8} - \epsilon ^{\alpha } \omega ^{\alpha } \rho ^{\alpha } (\eta _{s}^{\alpha })^*)}, \\&I_{s}^* = \frac{\tau ^{\alpha } (\varpi ^{\alpha } \gamma _{1}^{\alpha } + \sigma ^{\alpha } k_{5})E^*}{k_{4}k_{5}}, \\&I_{a}^* = \frac{\tau ^{\alpha } \varpi ^{\alpha } E^*}{k_{5}}, \\&I_{m}^* = \frac{\tau ^{\alpha } (\varpi ^{\alpha } \gamma _{2}^{\alpha } + k_{5}k_{6})E^*}{k_{5}k_{7}}, \\&R^* = \frac{\tau ^{\alpha }((\eta _{s}^{\alpha })^* + \mu ^{\alpha })((\eta _{s}^{\alpha })^* + k_{2})E^* \Phi + \Lambda ^{\alpha } k_{4}k_{5}k_{7}k_{8}}{k_{4}k_{5}k_{7}(((\eta _{s}^{\alpha })^* + k_{2})((\eta _{s}^{\alpha })^* + \mu ^{\alpha })k_{8} - \epsilon ^{\alpha } \omega ^{\alpha } \rho ^{\alpha } (\eta _{s}^{\alpha })^*)}, \end{aligned}$$

where

$$\begin{aligned} \Phi = (k_{7} \alpha _{1}^{\alpha } \varphi ^{\alpha } + \alpha _{3}^{\alpha }(\varphi ^{\alpha } \gamma _{2}^{\alpha } + k_{5}k_{6}))k_{4} + k_{7}\alpha _{2}^{\alpha }(\sigma ^{\alpha }k_{5} + \varphi ^{\alpha } \gamma _{1}^{\alpha }), \end{aligned}$$

37 $$\begin{aligned} (\eta _{s}^{\alpha })^* = \frac{\beta (I_{a}^* + \nu _{1}I_{s}^* + \nu _{2}I_{m}^*)}{N^*}, \end{aligned}$$

and

38 $$\begin{aligned} N^* = S^* + V^* + E + I_{s}^* + I_{a}^* + I_{m}^* + R^*. \end{aligned}$$

Equation (37) can now be written as

39 $$\begin{aligned} S^* + V^* + \left( 1 - \frac{\beta \nu _{1}}{(\eta _{s}^{\alpha })^*}\right) I_{s}^* + \left( 1 - \frac{\beta }{(\eta _{s}^{\alpha })^*}\right) I_{a}^* + \left( 1 - \frac{\beta \nu _{2}}{(\eta _{s}^{\alpha })^*}\right) I_{m}^* + R^* = 0. \end{aligned}$$

After simplifying (39), we obtained the following: $(\eta _{s}^{\alpha })^* = 0$ as one of the solutions (which corresponds to the DFE and the quadratic equation):

40 $$\begin{aligned} a_{0} ((\eta _{s}^{\alpha })^*)^{2} + a_{1} (\eta _{s}^{\alpha })^* + a_{2} = 0, \end{aligned}$$

where

41 $$\begin{aligned} a_{0}&= \big (\big (\varpi ^{\alpha } \big (k_{8} + \alpha _{1}^{\alpha } \big )k_{7} + \big (\varpi ^{\alpha } \gamma _{2}^{\alpha } + k_{5} k_{6} \big ) \big (\alpha _{3}^{\alpha } + k_{8} \big ) \big )k_{4} + k_{7} \big (\sigma ^{\alpha } k_{5} + \varphi ^{\alpha } \gamma _{1}^{\alpha } \big ) \big (\alpha _{2}^{\alpha } + k_{8} \big ) \big )\tau ^{\alpha } \\&\quad + k_{4} k_{5}k_{7}k_{8}, \\ a_{1}&= \big (1 - \epsilon ^{\alpha } \big ) \big (\omega ^{\alpha } \sigma ^{\alpha } \tau ^{\alpha } \alpha _{2}^{\alpha } k_{5}k_{7} + \omega ^{\alpha } \sigma ^{\alpha } \tau ^{\alpha } k_{5}k_{7}k_{8} + \omega ^{\alpha } \tau ^{\alpha } \varpi ^{\alpha } \alpha _{1}^{\alpha } k_{4}k_{7} + \omega ^{\alpha } \tau ^{\alpha } \varpi ^{\alpha } \alpha _{2}^{\alpha } \gamma _{1}^{\alpha } k_{7} \\&\quad + \omega ^{\alpha } \tau ^{\alpha } \varpi ^{\alpha } \alpha _{3}^{\alpha } \gamma _{2}^{\alpha } k_{4} + \omega ^{\alpha } \tau ^{\alpha } \varpi ^{\alpha } \gamma _{1}^{\alpha } k_{7}k_{8} + \omega ^{\alpha } \tau ^{\alpha } \varpi ^{\alpha } \gamma _{2}^{\alpha } k_{4}k_{8} + \omega ^{\alpha } \tau ^{\alpha } \varpi ^{\alpha } k_{4}k_{7}k_{8} + \omega ^{\alpha } \tau ^{\alpha } \alpha _{3}^{\alpha } k_{4}k_{5}k_{6} \\&\quad + \omega ^{\alpha } \tau ^{\alpha } k_{4}k_{5}k_{6}k_{8} + \omega ^{\alpha } k_{4}k_{5}k_{7}k_{8} \big ) - \beta ^{\alpha } \sigma ^{\alpha } \tau ^{\alpha } k_{5}k_{7}k_{8} \nu _{1}^{\alpha } - \beta ^{\alpha } \tau ^{\alpha } \varpi ^{\alpha } \gamma _{1}^{\alpha } k_{7}k_{8} \nu _{1}^{\alpha } - \beta ^{\alpha } \tau ^{\alpha } \varpi ^{\alpha } \gamma _{2}^{\alpha } k_{4}k_{8} \nu _{2}^{\alpha } \\&\quad - \beta ^{\alpha } \tau ^{\alpha } k_{4}k_{5}k_{6}k_{8} \nu _{2}^{\alpha } - \beta ^{\alpha } \tau ^{\alpha } \varpi ^{\alpha } k_{4}k_{7} k_{8} + \epsilon ^{\alpha } \omega ^{\alpha } k_{3}k_{4}k_{5}k_{7} + \mu ^{\alpha } \sigma ^{\alpha } \tau ^{\alpha } \alpha _{2}^{\alpha } k_{5} k_{7} + \mu ^{\alpha } \sigma ^{\alpha } \tau ^{\alpha } k_{5}k_{7}k_{8} \\&\quad + \mu ^{\alpha } \tau ^{\alpha } \varpi ^{\alpha } \alpha _{1}^{\alpha } k_{4}k_{7} + \mu ^{\alpha } \tau ^{\alpha } \varpi ^{\alpha } \alpha _{2}^{\alpha } \gamma _{1}^{\alpha } k_{7} + \mu ^{\alpha } \tau ^{\alpha } \varpi ^{\alpha } \alpha _{3}^{\alpha } \gamma _{2}^{\alpha }k_{4} + \mu ^{\alpha } \tau ^{\alpha } \varpi ^{\alpha } \gamma _{1}^{\alpha } k_{7}k_{8} + \mu ^{\alpha } \tau ^{\alpha } \varpi ^{\alpha } \gamma _{2}^{\alpha }k_{4}k_{8} \\&\quad + \mu ^{\alpha } \tau ^{\alpha } \varpi ^{\alpha } k_{4}k_{7}k_{8} + \mu ^{\alpha } \tau ^{\alpha } \alpha _{3}^{\alpha } k_{4}k_{5}k_{6} + \mu ^{\alpha } \tau ^{\alpha } k_{4}k_{5}k_{6}k_{8} + \mu ^{\alpha } k_{4}k_{5}k_{7}k_{8} + k_{3}k_{4}k_{5}k_{7}k_{8}, \\ a_{2}&= k_{2}k_{3}k_{4}k_{5}k_{7}k_{8} - \tau ^{\alpha } \beta ^{\alpha } k_{1} k_{8}\big ( \big (\varpi ^{\alpha } k_{7} + \nu _{2} \big (\varpi ^{\alpha } \gamma _{2}^{\alpha } + k_{5}k_{6} \big ) \big )k_{4} + k_{7} \nu _{1}^{\alpha } \big (\sigma ^{\alpha } k_{5} + \varpi ^{\alpha } \gamma _{1}^{\alpha } \big ) \big ) \\&= k_{2}k_{3}k_{4}k_{5}k_{7}k_{8} \big (1 - R_{eff} \big ). \end{aligned}$$

Therefore, by simplifying (40) and substituting into *EE*, we then obtain a positive *EE*: Hence, we get the following result:

##### Theorem 7

From (3):

1. $R_{eff} > 1$ or $a_{2} < 0$ implies a unique endemic equilibrium,
2. Also $a_{1} < 0$ and $R_{eff} = 1$ or $\Delta = a_{1}^{2}-4 a_{0}a_{2} = 0$ implies a unique endemic equilibrium,
3. $a_{1}< 0, R_{eff} < 1$ and $\Delta > 0$ implies two endemic equilibrium, and
4. no endemic equilibrium otherwise.

Since all the model parameters are positive it is obvious that $a_{0} > 0$ and the proof complies with the characteristics of quadratic equation roots. $a_{2}$ is either positive or negative depending on whether $R_{eff} < 1 \text { or } R_{eff} > 1$. Clearly, from the case (i) of Theorem 7 whenever $R_{eff} > 1$, (3) has a unique EE. From case (iii) of Theorem 7, we get a backward bifurcation, this is a scenario where stable DFE and stable EE coexist whenever $R_{eff} < 1$ (see,^66–68^ and references therein for discussions on bifurcation analysis). We verify the backward bifurcation (BB) in a similar manner in^69,70^ by first letting discriminant $a_{1}^2 - 4 a_{0} a_{2} = 0$ and simplifying for the critical value of $R_{eff}$, denoted by $R_{eff}^{c}$ and given by

42 $$\begin{aligned} R_{eff}^{c} = 1 - \frac{a_{1}^2}{4a_{0}k_{2}k_{3}k_{4}k_{5}k_{7}k_{8}}. \end{aligned}$$

**Figure 4:**
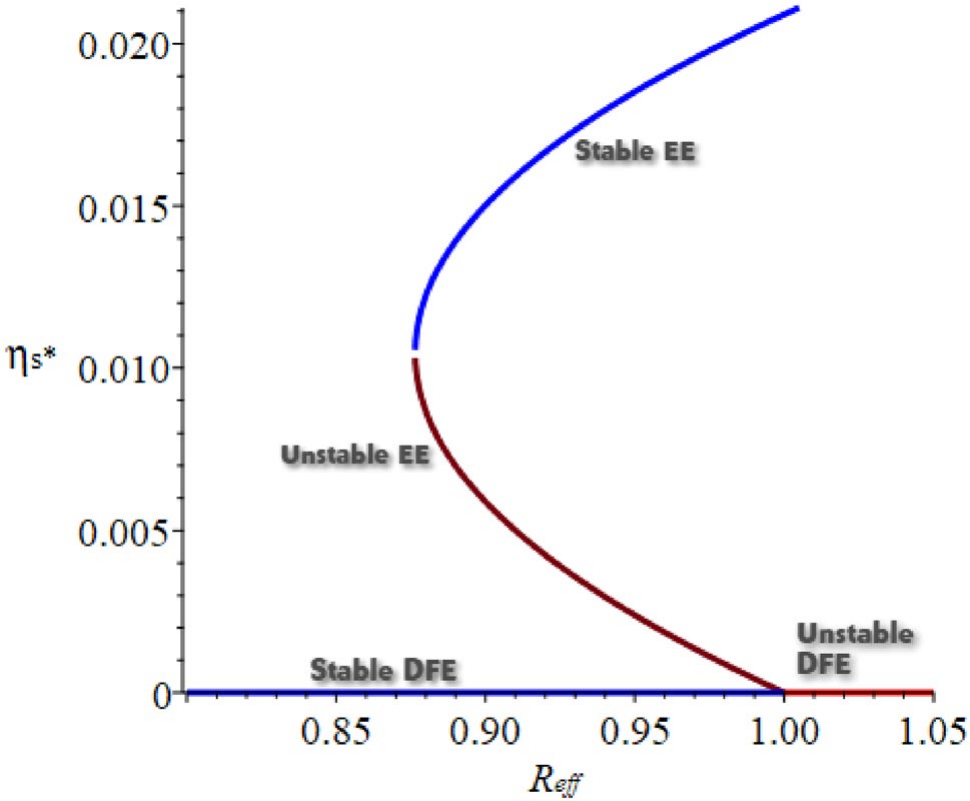
Bifurcation diagram of the model (3). So that, $a_{0} = 0.4849076636 \times 10^{-1}, a_{1} = -0.1013622972 \times 10^{-2}, a_{2} = 5.647464170 \times 10^{-7}, R_{eff}^{c} = 0.8764132312 \text {, and } R_{eff} = 0.9868237565 ({\text {that}}\,{\text{is}}, R_{eff}^{c}< R_{eff} < 1)$.

The BB would then occur for the values of $R_{eff}^{c}$ such that $R_{eff}^{c}< R_{eff} < 1$. It can be seen in Fig. 4, this is illustrated by simulating the model with the following set of parameter values. It is important to note that the parameters used in this illustration are chosen for just demonstration purposes. The parameters used are given in Table 3, with $\beta = 0.3888 \text {, } \omega = 0.01202643 \text {, } \epsilon = 0.3 \text {, } \nu _{1} = 0.3 \text {, } \nu _{2} = 0.1 \text {, } \varpi = 0.2 \text {, } \sigma = 0.899 \text {, } \gamma _{1} = 0.3 \text {, } \gamma _{2} = 0.2 \text {, } \delta _{1} = 0.028, \delta _{2} = 0.025 \text {, } \alpha _{3} = 0.1203 \text {, } \rho = 0.5 \text {, and } \alpha \in (0, 1]$. So that, $a_{0} = 0.4849076636 \times 10^{-1}, a_{1} = -0.1013622972 \times 10^{-2}, a_{2} = 5.647464170 \times 10^{-7}, R_{eff}^{c} = 0.8764132312 \text {, and } R_{eff} = 0.9868237565 (\text {that is}, R_{eff}^{c}< R_{eff} < 1)$.

#### Global stability analysis of the endemic equilibrium

From^71–73^, we have the following results:

##### Theorem 8

The unique endemic equilibrium is globally asymptomatically stable in ${\mathcal {D}}$ when $R_{eff} > 1$, provided that

43 $$\begin{aligned} \left( 1 - \frac{\eta _{s}^{\alpha }}{(\eta _{s}^{\alpha })^*} \right) \left( 1 - \frac{I_{s} (\eta _{s}^{\alpha })^*}{I_{s}^* \eta _{s}^{\alpha }} \right) \ge 0 \end{aligned}$$

and

44 $$\begin{aligned} \left( 1 - \frac{\eta _{s}^{\alpha }}{(\eta _{s}^{\alpha })^*} \right) \left( 1 - \frac{I_{m} (\eta _{s}^{\alpha })^*}{I_{m}^* \eta _{s}^{\alpha }} \right) \ge 0 \end{aligned}$$

are true.

See “Appendix” section for the proof.

## Numerical simulations of the model

Using COVID-19 data obtained from Thailand, we carried out various simulations to show the transmission dynamics of the disease, considering many scenarios.

### Numerical results

**Figure 5:**
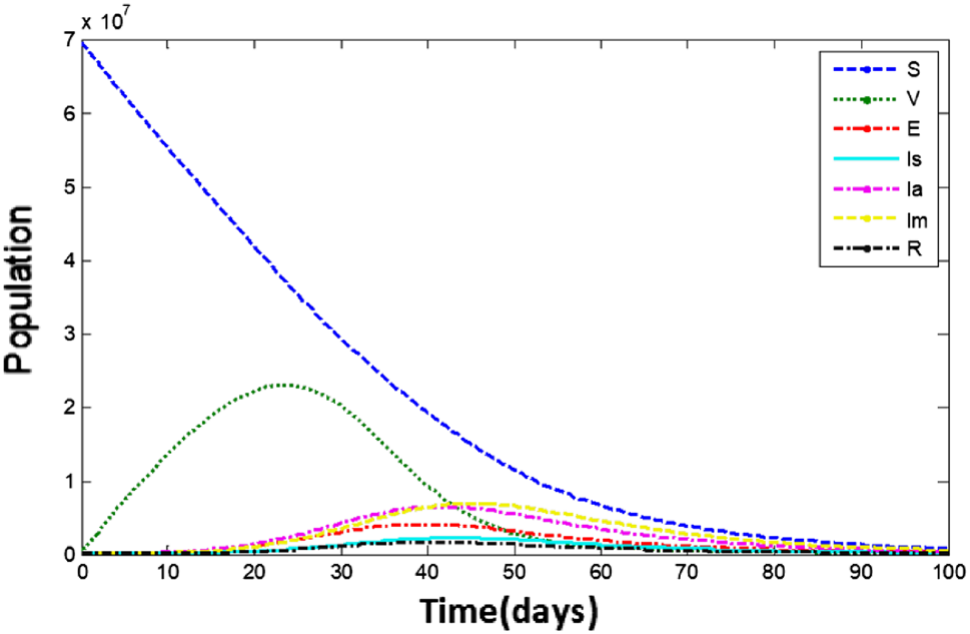
Dynamics of the whole population.

Figure 5 shows the time-series simulation results for (3).

**Figure 6:**
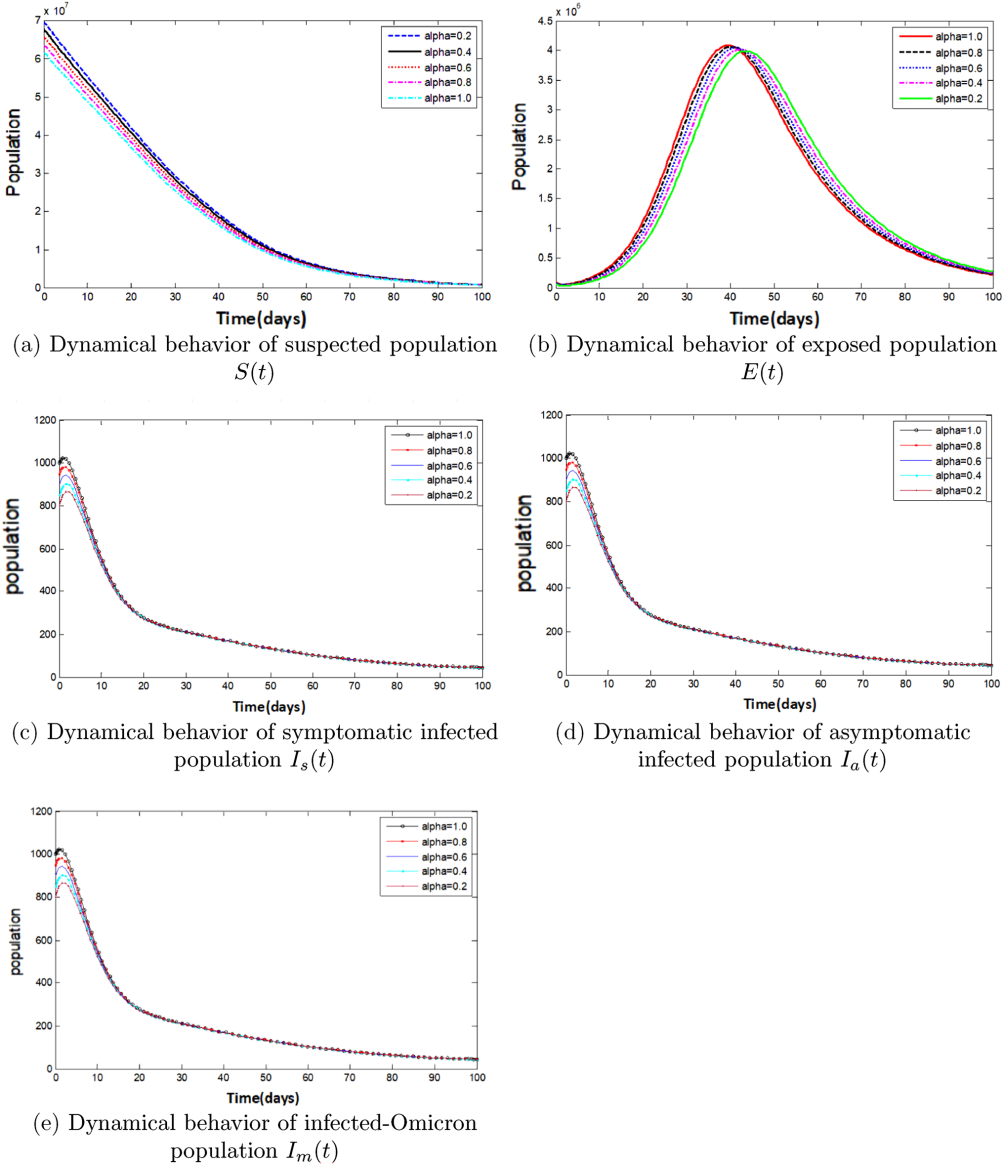
Graphs for the nature of each state variable for the Caputo version of the fractional model at different values of $\alpha$.

Next, using the Caputo operator $(\alpha )$, numerical simulations based on the fractional model are presented. As a result, (11) is numerically solved as described in^74^ using the biological parameter values presented in Table 3. Figure 6 shows the simulation results for varying $\alpha$ values of the state variables over time.

Figure 6a shows that the number of people who are susceptible (i.e., the number of people who are not immune to COVID-19) decreases over time. This is because as more people become infected or vaccinated, they become immune and reduce the pool of susceptible people. Figure 6b shows the exposed population for various values of $\alpha$. The graph shows that this population reached a maximum peak and then began to decline. The peak in the graph suggests a high level of transmission, where the virus rapidly spreads among susceptible people. However, as more people are vaccinated or recover from the infection, the exposed population starts to decline. This decline indicates that vaccination programs and other interventions are effective in reducing viral transmissions. Figure 6c–e show the populations of infected symptomatic, asymptomatic, and Omicron groups, respectively, for different values of $\alpha$. The graphs indicate that the interventions implemented in the model, such as NPIs and vaccines, were effective in reducing the number of infections in all three groups. This suggests that these measures can control the spread of the virus and mitigate the severity of the disease, regardless of whether individuals show symptoms or are infected with the Omicron variant. However, it is important to note that the asymptomatic group can still transmit the virus to others; therefore, they should be considered as a potential source of transmission.

**Figure 7:**
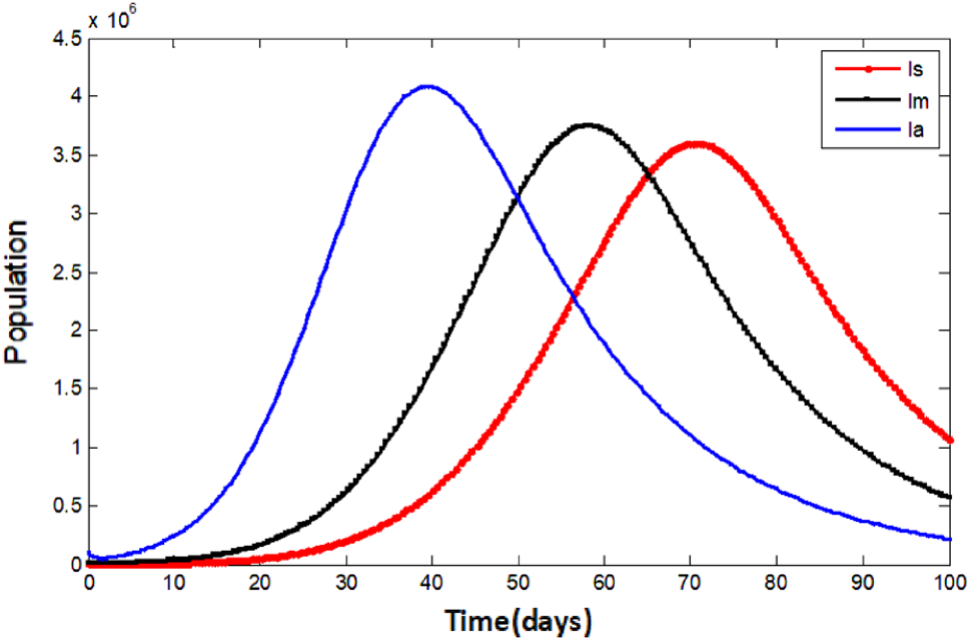
Comparing the three Infected population classes.

Figure 7 compares the three infected populations (symptomatic, asymptomatic, and Omicron-infected) and provides a comprehensive visual representation of the simulations. The graph enables for direct comparison of the trends and magnitudes of the three populations. These observations signal that the Omicron variant is more contagious and spreads the disease more rapidly than the other COVID-19 variants. It is also important to acknowledge that the asymptomatic infected population can contribute to the transmission of the virus despite the absence of symptoms. Consequently, controlling the spread of both symptomatic and asymptomatic infections is crucial for preventing further propagation of the virus.

**Figure 8:**
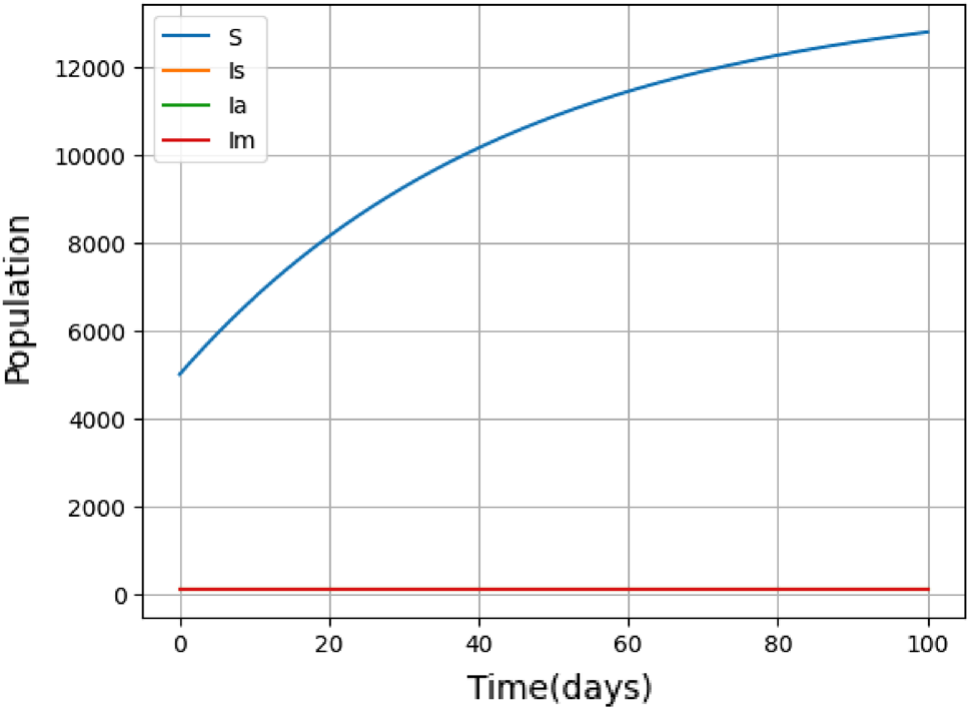
DFE of Susceptible (*S*) and Infected $(I_{s}, I_{a}, I_{m})$ population.

**Figure 9:**
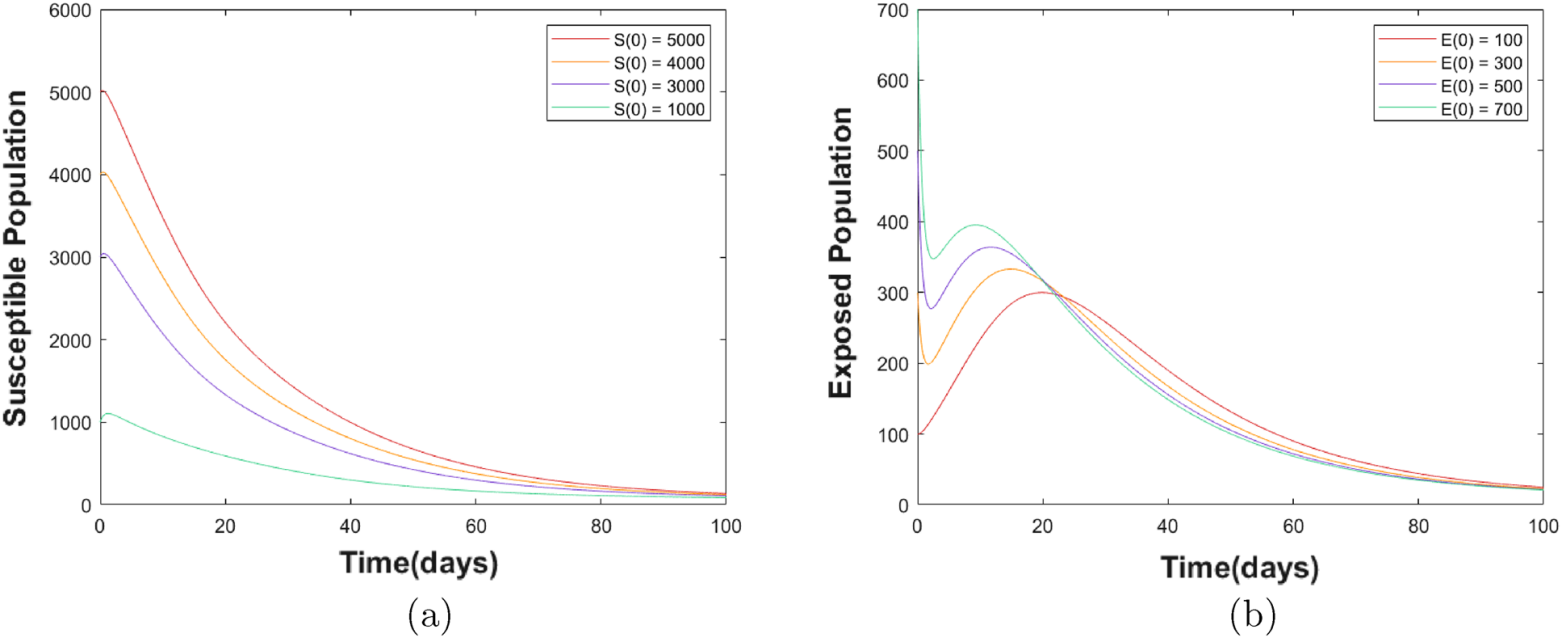
(**a**) Endemic equilibrium for susceptible population S(t). (**b**) Endemic equilibrium for exposed population E(t).

Figure 8 shows the global stability of the disease-free equilibrium (DFE), where no one is infected and the disease cannot spread. The figure shows that the infected population always stays at zero over time, whereas the susceptible population increases to attain a steady state. Figure 9 illustrates the dynamic shifts in the susceptible and exposed populations as time progressed, specifically when the infection became endemic within the population. This captures fluctuating levels of susceptibility and exposure, highlighting the evolving nature of the impact of the disease on the population over time.

### Vaccine intervention and global sensitivity analysis

The use of the COVID-19 vaccine helps reduce the spread of the virus^51^. In this section, we evaluate the impact of the vaccine and its effectiveness on the reproduction number. We define the reproduction number in the absence of a vaccine as a basic reproduction number

45 $$\begin{aligned} R_{0} = \frac{\beta ^{\alpha } \tau ^{\alpha } \big ( \big (\varpi ^{\alpha } k_{7} + \nu _{2}^{\alpha } \big (\varpi ^{\alpha } \gamma _{2}^{\alpha } + k_{5} k_{6} \big ) \big )k_{4}+ k_{7} \nu _{1}^{\alpha } \big (\sigma ^{\alpha } k_{5} + \varpi ^{\alpha } \gamma _{1}^{\alpha } \big ) \big )}{k_{3}k_{4}k_{5}k_{7}}. \end{aligned}$$

Using Eqs. (31) and (45), we get:

46 $$\begin{aligned} R_{eff} - R_{0} = \frac{k_{1} \beta ^{\alpha } \tau ^{\alpha } \big (k_{1} - k_{2}\big ) \big ( \big (\varpi ^{\alpha } k_{7} + \nu _{2}^{\alpha } \big (\varpi ^{\alpha } \gamma _{2}^{\alpha } + k_{5} k_{6} \big ) \big )k_{4}+ k_{7} \nu _{1}^{\alpha } \big (\sigma ^{\alpha } k_{5} + \varpi ^{\alpha } \gamma _{1}^{\alpha } \big ) \big )}{k_{2}k_{3}k_{4}k_{5}k_{7}}. \end{aligned}$$

Note that $k_{1} - k_{2} = -\omega ^{\alpha } \epsilon ^{\alpha }$, hence $R_{eff} - R_{0}$ is strictly negative. The implication of this is that using the vaccine effectively will have a strong impact on the reduction of the spread of all COVID-19 variants including Omicron. Using the fitted and estimated parameters from Table 3, we can also estimate the basic reproduction number and effective reproduction number as $R_{0} = 1.828794173 \text { and } R_{eff} = 0.9159273511$ respectively.

#### Global sensitivity analysis using partial rank correlation coefficients

**Figure 10:**
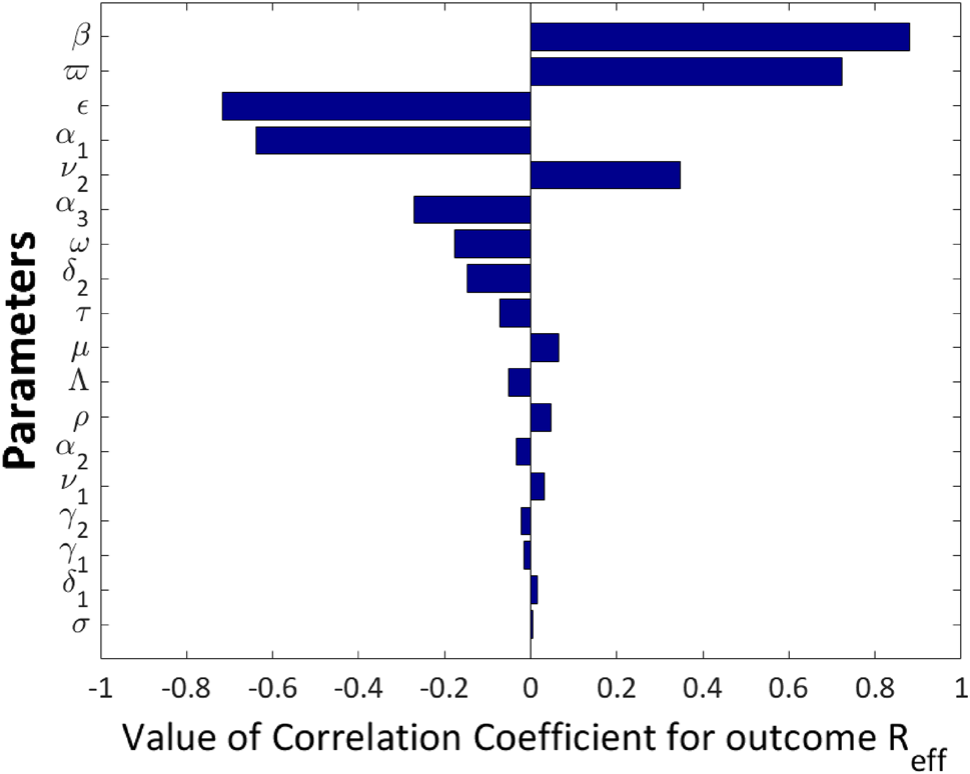
Tornado plot showing the sensitivities of the model parameters affecting the effective reproduction number $R_{eff}$.

In epidemic modeling, errors occur when attempting to estimate parameter values for the model. Often there is a tendency for these parameters to be based on incomplete or limited data, which can lead to estimates that are inaccurately reflective of the actual population. In addition, the precise value of several parameters that are being evaluated is frequently uncertain. Variations between groups or areas, as well as personal circumstances that could not be considered, may lead to inaccuracies. Even with sufficient data and accuracy checks built in, parameter uncertainty is still probably caused by time-changing conditions within a given population or abrupt alterations because of unforeseeable occurrences like natural disasters or civil unrest. For these reasons, it’s crucial to carry out sampling and sensitivity analysis to identify the variables that significantly affect model output. The Sampling and Sensitivity Analysis Tool (SaSAT) is a software tool developed for such purposes (see^75^). In our model, 18 different COVID-19 epidemiological parameters, whose values varied from other research and model fitting to data were used to regulate the effective reproduction number. Each of these parameters had baseline values and ranges assigned to them following^66^. In order to create a 1000 by 18 matrix with each row defining a different parameter set, we utilized Latin hypercube sampling (LHS) to draw 1000 samples for each of the parameters. The effective reproduction numbers were calculated using the parameter sets, and the statistical contribution of each parameter to the reproduction numbers was then described using the partial rank correlation coefficient (PRCC). Figure 10 shows the tornado plot of the results.

The top five most sensitive parameters affecting the $R_{eff}$ are $\beta , \varpi , \epsilon , \alpha _{1} \text {, and } \nu _2$ in that order, as shown in Figure 10. To reduce the value of $R_{eff}$, we need to reduce $\beta , \varpi \text {, and } \nu _{2}$ or increase the values of $\epsilon \text { and } \alpha _{1}$. It is important to note that a faster decline in the value of $R_{eff}$ will result from simultaneously increasing the values of parameters with negative PRCC values and decreasing the values of parameters with positive PRCC values.

**Figure 11:**
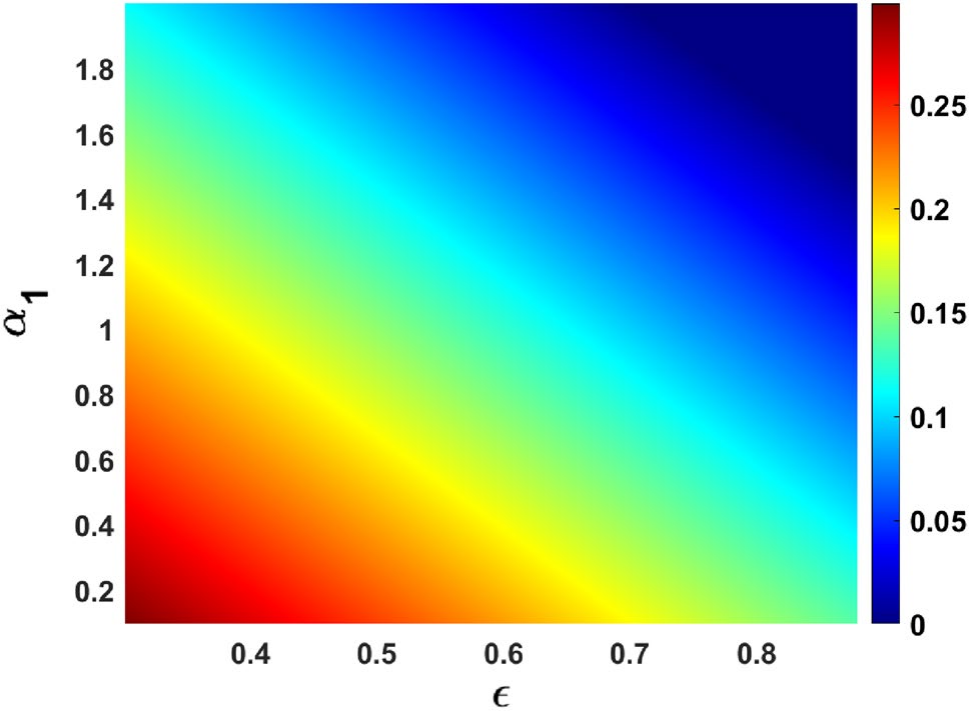
response surface plot of $R_{eff}$ with respect to $\alpha _{1} \text { versus } \epsilon$.

Vaccines with a high level of efficacy have the potential to reduce the number of secondary infections in the community to a considerable extent. The graphical representation (response surface plot) of $R_{eff}$ in the parameter space $(\epsilon , \alpha _{1})$ see Fig. 11, which tends to suggest that the impact of the vaccine effectiveness is similar to the effect of recovery of the asymptomatic infected individuals.

**Figure 12:**
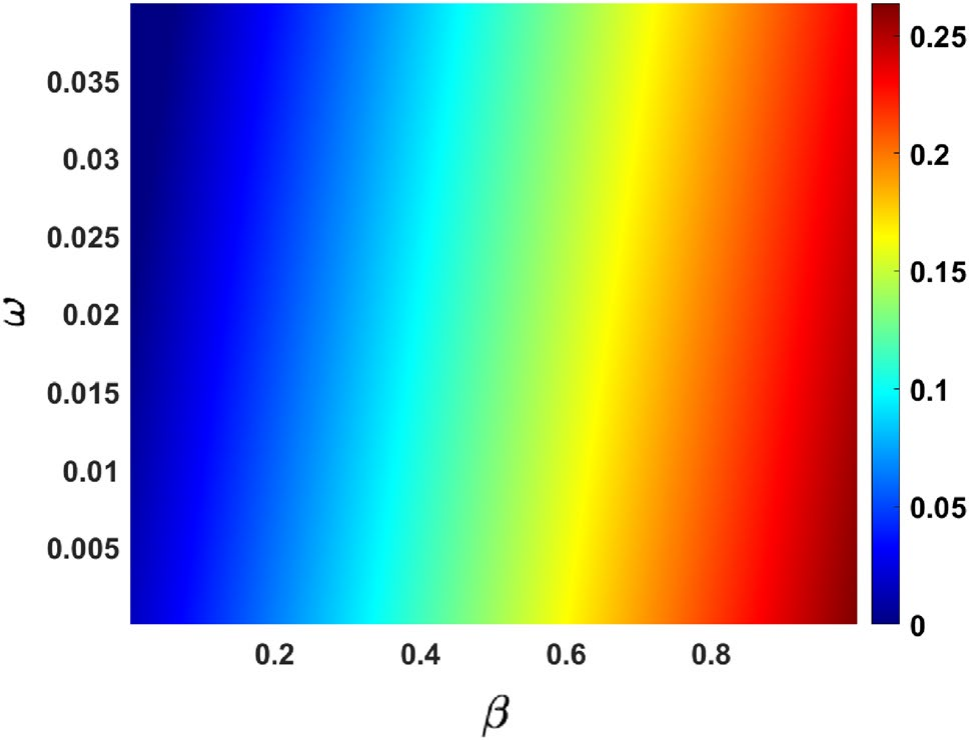
response surface plot of $R_{eff}$ with respect to $\beta \text { versus } \omega$.

**Figure 13:**
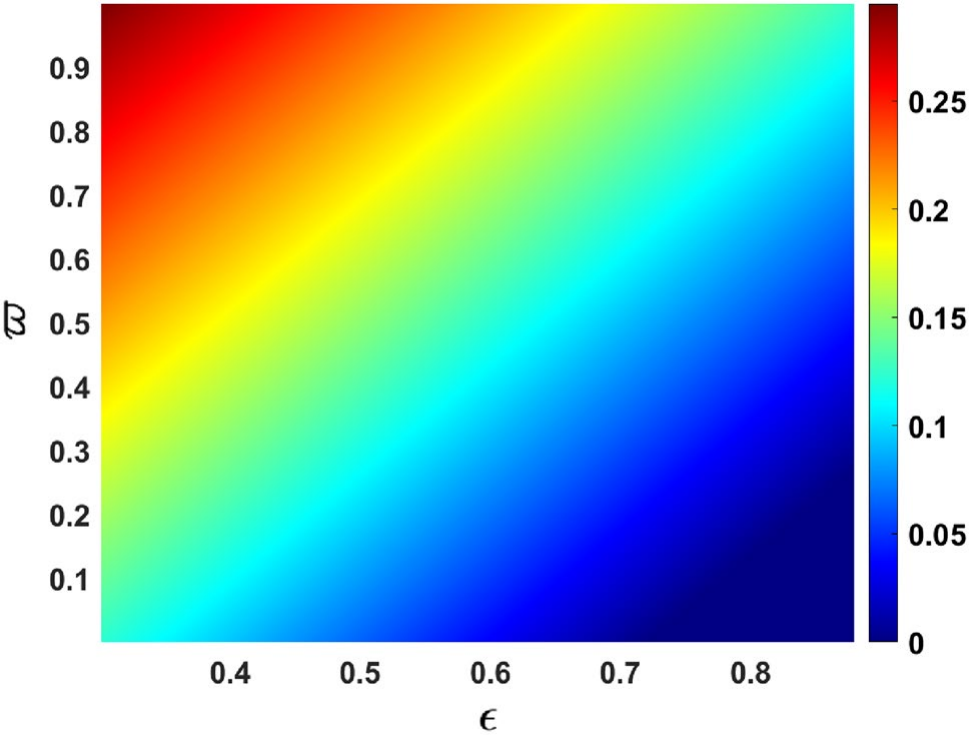
response surface plot of $R_{eff}$ with respect to $\varpi \text { versus } \epsilon$.

Figure 12 depicts the impact of the effective contact rate $\beta$ vs. vaccination rate of the susceptible individuals $\omega$ on the effective reproduction number. Figure 13 depicts the impact of the rate in progression from exposed individuals $\varpi$ vs. infection reduction of the vaccinated individuals due to the vaccine effectiveness $\epsilon$.

## Discussion

Mathematical models provide efficient techniques for investigating how COVID-19 works and its potential behavior over time. Researchers and governments are using these models to predict how the virus will spread and help them make decisions about mitigation strategies, resource allocation, vaccine development, public health messaging, and other aspects of dealing with the virus.

In this study, we modeled the transmission and spread of COVID-19 by considering vaccination and vaccine effectiveness. Our model indicated that vaccination (a successful vaccine against COVID-19) should not be seen as the only solution that will eradicate this disease but rather as a valuable tool for containing its spread and reducing severe illnesses within populations. To attain true victory against this pandemic, attention must be given to the recovery of asymptomatic COVID-19-infected individuals. These individuals pose a unique challenge in controlling the spread of the disease. In other words, as a first step, and perhaps the most important, is to encourage individuals to get tested if they have any reason to believe that they have been exposed to someone who is recently diagnosed with COVID-19 or someone exhibiting symptoms.

Furthermore, it is crucial that these individuals follow up with medical personnel and stay in contact regarding any further instructions that they may require. If there are any new symptoms that appear in the future, they should notify the medical personnel as soon as possible, so that they can get proper care and advice if necessary. Additionally, they should also practice proper hygiene measures, including washing their hands regularly and maintaining proper social distancing while out in public at all times.

From the results of our PRCC calculations, we found that the top five parameters that have the most influence on the disease transmission dynamics are effective contact rate, the rate of progression from exposed to asymptomatic infected individuals, infection reduction due to vaccine effectiveness, the recovery rate of asymptomatic infected individuals, and infectiousness of omicron individuals. These do not fully support the finding in^26^, which showed that the top 5 parameters of their model were the contact rate, infections of the omicron-infected individuals, incubation period, recovery rate of the asymptomatic infected individuals, and the rate of flow to omicron infected individuals.

The sensitivity analysis results revealed that significantly improving vaccine effectiveness and high recovery rate of the asymptomatic individuals can make the disease be eradicated. The current study agrees with the work of many authors that reported a significant decrease in COVID-19 transmission (see, for example,^76^).

A response surface plot of the effective reproduction number, as a function of the recovery rate of the asymptomatic infected individuals and a fraction of the reduction of infections due to vaccine effectiveness, is depicted in Fig. 12. It follows from this figure if both parameters are increased, that could possibly eradicate the spread of COVID-19. Another parameter in our model that can be targeted for interventions is the vaccination rate of susceptible individuals. According to official government data, more than half of Thailand’s population has been vaccinated for the virus. An increase in this parameter can lead to a reduction in the effective reproduction number. The appearance of $(\omega )$ as one of the top six most sensitive parameters is quite significant because it supports the work of^27^.

## Conclusions

In this paper, we presented a comprehensive mathematical analysis of the transmission dynamics of COVID-19 infections in Thailand, taking into account the different clinical manifestations of the disease and the emergence of the omicron variant. We developed an $S V E I_{s} I_{a} I_{m} R$ model that captures the interactions among susceptible, vaccinated, exposed, symptomatic, asymptomatic, and omicron-infected individuals. The following are some of the main findings of this study:

(1): We calculated the effective reproduction number $(R_{eff})$ and established the conditions for both local and global stability of the equilibrium points for the model (3).
(2): We also showed that the model exhibits backward bifurcation at $R_{eff} = 1$ which brings about a sudden change from a stable equilibrium to an unstable one.
(3): To determine the most crucial parameters that control the dynamics of COVID-19 transmissions, we performed a global sensitivity analysis utilizing Latin Hypercube Sampling and Partial Rank Correlation Coefficient and we discovered the most important parameters in controlling this pandemic are effective contact rate, the rate of progression from exposed to asymptomatic infected individuals, infection reduction due to vaccine effectiveness, the recovery rate of asymptomatic infected individuals, and infectiousness of omicron individuals.
(4): To demonstrate some of the aforementioned theoretical findings, numerical simulations are carried out using the fitted parameters to Thailand data or cited from existing literature.

Our study provides valuable insights into the epidemiology and control of COVID-19 in Thailand. Based on our findings, we suggest that public health authorities and policymakers should prioritize increasing vaccination coverage, enhancing testing and tracing capacities, enforcing social distancing and mask wearing measures, and monitoring the emergence and spread of new variants. These interventions can help reduce the transmission potential of COVID-19 and prevent future outbreaks.

## Data Availability

The datasets used and/or analyzed during the current study are available from the corresponding author upon reasonable request.

## The proof of theorem 8

### Proof

We use the approach in^71–73^ to establish the proof. If we first consider a Lyapunov function:

47 $$\begin{aligned} F_{2}&= J_{1} \left( S - S^* - S^* \ln \frac{S}{S^*} \right) + J_{2} \left( V - V^* - V^* \ln \frac{V}{V^*} \right) + J_{3} \left( E - E^* - E^* \ln \frac{E}{E^*} \right) \\&\quad + J_{4} \left( I_{s} - I_{s}^* - I_{s}^* \ln \frac{I_{s}}{I_{s}^*} \right) + J_{5} \left( I_{a} - I_{a}^* - I_{a}^* \ln \frac{I_{a}}{I_{a}^*} \right) + J_{6} \left( I_{m} - I_{m}^* - I_{m}^* \ln \frac{I_{m}}{I_{m}^*} \right) . \end{aligned}$$

where $J_{i} > 0 \text { }(i = 1, 2, 3, 4, 5, 6)$ are constants to be determined. It is easy to see that $F_{2} \ge 0 \text { for all } S, V, E, I_{s}, I_{a}, I_{m} > 0, \text { and } F_{2} = 0 \iff (S, V, E, I_{s}, I_{a}, I_{m}) = (S^*, V^*, E^*, I_{s}^*, I_{a}^*, I_{m}^*)$. The Lyapunov function has a derivative

A-1 $$\begin{aligned} \dot{F_{2}}(t)&= J_{1} \left( 1 - \frac{S^*}{S} \right) \dot{S} + J_{2} \left( 1 - \frac{V^*}{V} \right) \dot{V} + J_{3} \left( 1 - \frac{E^*}{E} \right) \dot{E} \\&\quad + J_{4} \left( 1 - \frac{I_{s}^*}{I_{s}} \right) \dot{I_{s}} + J_{5} \left( 1 - \frac{I_{a}^*}{I_{a}} \right) \dot{I_{a}} + J_{6} \left( 1 - \frac{I_{m}^*}{I_{m}} \right) \dot{I_{m}} . \end{aligned}$$

By direct computation from (A-1), we have

A-2 $$\begin{aligned} J_{1} \left( 1 - \frac{S^*}{S} \right) \dot{S}&= J_{1} \left( 1 - \frac{S^*}{S} \right) (\Lambda ^{\alpha } + \rho ^{\alpha } R - (\eta _{s}^{\alpha } + k_{2})S) \\&= J_{1} \left( 1 - \frac{S^*}{S} \right) (\Lambda ^{\alpha } + \rho ^{\alpha } R - \eta _{s}^{\alpha } S - k_{2}S) \\&= J_{1} \left( 1 - \frac{S^*}{S} \right) ((\eta _{s}^{\alpha })^* S^* + k_{2}S^* - \eta _{s}^{\alpha } S - k_{2}S) \\&= J_{1} \left( 1 - \frac{S^*}{S} \right) \left( (\eta _{s}^{\alpha })^* S^* \left( 1 - \frac{\eta _{s}^{\alpha } S}{(\eta _{s}^{\alpha })^* S^*}\right) - k_{2}(S - S^*)\right) \\&= J_{1} (\eta _{s}^{\alpha })^* S^* \left( 1 - \frac{S^*}{S} \right) \left( 1 - \frac{\eta _{s}^{\alpha } S}{(\eta _{s}^{\alpha })^* S^*}\right) - J_{1} k_{2} \left( 1 - \frac{S^*}{S} \right) (S - S^*) \\&= J_{1} (\eta _{s}^{\alpha })^* S^* \left( 1 - \frac{S^*}{S} \right) \left( 1 - \frac{\eta _{s}^{\alpha } S}{(\eta _{s}^{\alpha })^* S^*}\right) - J_{1} k_{2} \frac{(S - S^*)^2}{S} \\&\le J_{1} (\eta _{s}^{\alpha })^* S^* \left( 1 - \frac{S^*}{S} \right) \left( 1 - \frac{\eta _{s}^{\alpha } S}{(\eta _{s}^{\alpha })^* S^*}\right) \\&= J_{1} (\eta _{s}^{\alpha })^* S^* \left( 1 - \frac{\eta _{s}^{\alpha } S}{(\eta _{s}^{\alpha })^* S^*} - \frac{S^*}{S} + \frac{\eta _{s}^{\alpha }}{(\eta _{s}^{\alpha })^*}\right) , \end{aligned}$$

and

A-3 $$\begin{aligned} J_{2} \left( 1 - \frac{V^*}{V} \right) \dot{V}&= J_{2} \left( 1 - \frac{V^*}{V} \right) (\omega ^{\alpha } S - (\eta _{s}^{\alpha } + \mu ^{\alpha })V) \\&= J_{2} \left( 1 - \frac{V^*}{V} \right) (\omega ^{\alpha } S - \eta _{s}^{\alpha } V - \mu ^{\alpha } V) \\&= J_{2} \left( 1 - \frac{V^*}{V} \right) ((\eta _{s}^{\alpha })^* V^* + \mu ^{\alpha } V^* - \eta _{s}^{\alpha } V - \mu ^{\alpha } V) \\&= J_{2} \left( 1 - \frac{V^*}{V} \right) \left( (\eta _{s}^{\alpha })^* V^* \left( 1 - \frac{\eta _{s}^{\alpha } V}{(\eta _{s}^{\alpha })^* V^*}\right) - \mu (V - V^*)\right) \\&= J_{2} (\eta _{s}^{\alpha })^* V^* \left( 1 - \frac{V^*}{V} \right) \left( 1 - \frac{\eta _{s}^{\alpha } V}{(\eta _{s}^{\alpha })^* V^*}\right) - J_{2} \mu \left( 1 - \frac{V^*}{V} \right) (V - V^*) \\&= J_{2} (\eta _{s}^{\alpha })^* V^* \left( 1 - \frac{V^*}{S} \right) \left( 1 - \frac{\eta _{s}^{\alpha } V}{(\eta _{s}^{\alpha })^* V^*}\right) - J_{2} g_{1} \frac{(V - V^*)^2}{V} \\&\le J_{2} (\eta _{s}^{\alpha })^* V^* \left( 1 - \frac{V^*}{V} \right) \left( 1 - \frac{\eta _{s}^{\alpha } V}{(\eta _{s}^{\alpha })^* V^*}\right) \\&= J_{2} (\eta _{s}^{\alpha })^* V^* \left( 1 - \frac{\eta _{s}^{\alpha } V}{(\eta _{s}^{\alpha })^* V^*} - \frac{V^*}{V} + \frac{(\eta _{s}^{\alpha })}{(\eta _{s}^{\alpha })^*}\right) , \end{aligned}$$

and

$$\begin{aligned} J_{3} \left( 1 - \frac{E^*}{E} \right) \dot{E} = J_{3} \left( 1 - \frac{E^*}{E} \right) (\eta _{s}^{\alpha } S + (1 - \epsilon ^{\alpha })\eta _{s}^{\alpha } V - k_{3} E) \end{aligned}$$

Let $$a = (1 - \epsilon ^{\alpha })$$

A-4 $$\begin{aligned} J_{3} \left( 1 - \frac{E^*}{E} \right) \dot{E}&= J_{3} \left( 1 - \frac{E^*}{E} \right) (\eta _{s}^{\alpha } S + a \eta _{s}^{\alpha } V - k_{3} E) \\&= J_{3} \left( 1 - \frac{E^*}{E} \right) \left( \eta _{s}^{\alpha } S + a \eta _{s}^{\alpha } V - \left( (\eta _{s}^{\alpha })^* S^*\frac{E}{E^*} + a (\eta _{s}^{\alpha })^* V^*\frac{E}{E^*}\right) \right) \\&= J_{3} \left( 1 - \frac{E^*}{E} \right) \left( \eta _{s}^{\alpha } S - (\eta _{s}^{\alpha })^* S^*\frac{E}{E^*} + a \eta _{s}^{\alpha } V - a (\eta _{s}^{\alpha })^* V^*\frac{E}{E^*} \right) \\&= J_{3} (\eta _{s}^{\alpha })^* S^* \left( 1 - \frac{E^*}{E} \right) \left( \frac{\eta _{s}^{\alpha } S}{(\eta _{s}^{\alpha })^* S^*} - \frac{E}{E^*} \right) \\&\quad + J_{3} a (\eta _{s}^{\alpha })^* V^* \left( 1 - \frac{E^*}{E} \right) \left( \frac{\eta _{s}^{\alpha } V}{(\eta _{s}^{\alpha })^* V^*} - \frac{E}{E^*} \right) \\&= J_{3} (\eta _{s}^{\alpha })^* S^* \left( \frac{\eta _{s}^{\alpha } S}{(\eta _{s}^{\alpha })^* S^*} - \frac{E}{E^*} - \frac{\eta _{s}^{\alpha } S E^*}{(\eta _{s}^{\alpha })^* S^* E} + 1 \right) \\&\quad + J_{3} a (\eta _{s}^{\alpha })^* V^* \left( \frac{\eta _{s}^{\alpha } V}{(\eta _{s}^{\alpha })^* V^*} - \frac{E}{E^*} - \frac{\eta _{s}^{\alpha } V E^*}{(\eta _{s}^{\alpha })^* V^* E} + 1 \right) , \end{aligned}$$

and

A-5 $$\begin{aligned} J_{4} \left( 1 - \frac{I_{s}^*}{I_{s}} \right) \dot{I_{s}}&= J_{4} \left( 1 - \frac{I_{s}^*}{I_{s}} \right) (\sigma ^{\alpha } \tau ^{\alpha } E + \gamma _{1}^{\alpha } I_{a} - k_{4} I_{s}) \\&= J_{4} \left( 1 - \frac{I_{s}^*}{I_{s}} \right) \left( \sigma ^{\alpha } \tau ^{\alpha } E + \gamma _{1}^{\alpha } I_{a} - \left( \frac{\sigma ^{\alpha } \tau ^{\alpha } E^*}{I_{s}^*} + \frac{\gamma _{1}^{\alpha } I_{a}^*}{I_{s}^*} \right) I_{s}\right) \\&= J_{4} \left( 1 - \frac{I_{s}^*}{I_{s}} \right) \left( \sigma ^{\alpha } \tau ^{\alpha } E - \frac{\sigma ^{\alpha } \tau ^{\alpha } E^* I_{s}}{I_{s}^*} + \gamma _{1}^{\alpha } I_{a} - \frac{\gamma _{1}^{\alpha } I_{a}^* I_{s}}{I_{s}^*}\right) \\&= J_{4} \sigma ^{\alpha } \tau ^{\alpha } E^* \left( 1 - \frac{I_{s}^*}{I_{s}} \right) \left( \frac{E}{E^*} - \frac{I_{s}}{I_{s}^*} \right) + J_{4} \gamma _{1}^{\alpha } I_{a}^* \left( 1 - \frac{I_{s}^*}{I_{s}} \right) \left( \frac{I_{a}}{I_{a}^*} - \frac{I_{s}}{I_{s}^*} \right) \\&= J_{4} \sigma ^{\alpha } \tau ^{\alpha } E^* \left( \frac{E}{E^*} - \frac{I_{s}}{I_{s}^*} - \frac{E I_{s}^*}{E^* I_{s}} + 1\right) + J_{4} \gamma _{1}^{\alpha } I_{a}^* \left( \frac{I_{a}}{I_{a}^*} - \frac{I_{s}}{I_{s}^*} - \frac{I_{a} I_{s}^*}{I_{a}^* I_{s}} + 1\right) , \end{aligned}$$

and

A-6 $$\begin{aligned} J_{5} \left( 1 - \frac{I_{a}^*}{I_{a}} \right) \dot{I_{a}}&= J_{5} \left( 1 - \frac{I_{a}^*}{I_{a}} \right) (\varpi ^{\alpha } \tau ^{\alpha } E - k_{5} I_{a}) \\&= J_{5} \left( 1 - \frac{I_{a}^*}{I_{a}} \right) \left( \varpi ^{\alpha } \tau ^{\alpha } E - \frac{\varpi ^{\alpha } \tau ^{\alpha } E^* I_{a}}{I_{a}^*} \right) \\&= J_{5} \varpi ^{\alpha } \tau ^{\alpha } E^* \left( 1 - \frac{I_{a}^*}{I_{a}} \right) \left( \frac{E}{E^*} - \frac{ I_{a}}{I_{a}^*} \right) \\&= J_{5} \varpi ^{\alpha } \tau ^{\alpha } E^* \left( \frac{E}{E^*} - \frac{ I_{a}}{I_{a}^*} - \frac{E I_{a}^*}{E^* I_{a}} + 1 \right) \end{aligned}$$

and

A-7 $$\begin{aligned} J_{6} \left( 1 - \frac{I_{m}^*}{I_{m}} \right) \dot{I_{m}}&= J_{6} \left( 1 - \frac{I_{m}^*}{I_{m}} \right) (k_{6} \tau ^{\alpha } E + \gamma _{2}^{\alpha } I_{a} - k_{7} I_{m}) \\&= J_{6} \left( 1 - \frac{I_{m}^*}{I_{m}} \right) \left( k_{6} \tau ^{\alpha } E + \gamma _{2}^{\alpha } I_{a} - \left( \frac{k_{6} \tau ^{\alpha } E^*}{I_{m}^*} + \frac{\gamma _{2}^{\alpha } I_{a}^*}{I_{m}^*} \right) I_{m}\right) \\&= J_{6} \left( 1 - \frac{I_{m}^*}{I_{m}} \right) \left( k_{6} \tau ^{\alpha } E - \frac{k_{6} \tau ^{\alpha } E^* I_{m}}{I_{m}^*} + \gamma _{2}^{\alpha } I_{a} - \frac{\gamma _{2}^{\alpha } I_{a}^* I_{m}}{I_{m}^*}\right) \\&= J_{6} k_{6} \tau ^{\alpha } E^* \left( 1 - \frac{I_{m}^*}{I_{m}} \right) \left( \frac{E}{E^*} - \frac{I_{m}}{I_{m}^*} \right) + J_{6} \gamma _{2}^{\alpha } I_{a}^* \left( 1 - \frac{I_{m}^*}{I_{m}} \right) \left( \frac{I_{a}}{I_{a}^*} - \frac{I_{m}}{I_{m}^*} \right) \\&= J_{6} k_{6} \tau ^{\alpha } E^* \left( 
\frac{E}{E^*} - \frac{I_{m}}{I_{m}^*} - \frac{E I_{m}^*}{E^* I_{m}} + 1\right) + J_{6} \gamma _{2}^{\alpha } I_{a}^* \left( \frac{I_{a}}{I_{a}^*} - \frac{I_{m}}{I_{m}^*} - \frac{I_{a} I_{m}^*}{I_{a}^* I_{m}} + 1\right) . \end{aligned}$$

Substituting $J_{1} = J_{3} = 1, J_{2} = a, J_{4} = \frac{(\eta _{s}^{\alpha })^{*} S^{*}}{(\sigma ^{\alpha } \tau ^{\alpha } E^{*} + \gamma _{1}^{\alpha } I_{a}^{*})}, J_{5} = \frac{(\eta _{s}^{\alpha })^{*} I_{a}^{*} G_{1}}{\varpi ^{\alpha } \tau ^{\alpha } G_{2}}, \text { and } J_{6} = \frac{a (\eta _{s}^{\alpha })^{*} V^{*}}{(\tau ^{\alpha } k_{5} E^{*} + \gamma _{2}^{\alpha } I_{a}^{*})}$, and (A-2)–(A-7) into (A-1), where

$$G_{1} = S^{*}(\tau ^{\alpha } \gamma _{1}^{\alpha } k_{6} E^{*} + \gamma _{1}^{\alpha } \gamma _{2}^{\alpha } I_{a}^{*}) + a V^{*}(\sigma ^{\alpha } \tau ^{\alpha } \gamma _{2}^{\alpha } E^{*} + \gamma _{1}^{\alpha } \gamma _{2}^{\alpha } I_{a}^{*})$$

$$G_{2} = (\tau ^{\alpha } k_{6} E^{*} + \gamma _{2}^{\alpha } I_{a}^{*})(\sigma ^{\alpha } \tau ^{\alpha } E^{*} + \gamma _{1}^{\alpha } I^{*})$$

we have

A-8 $$\begin{aligned} \dot{F_{2}}(t)&\le (\eta _{s}^{\alpha })^*S^* \left( 2 - \frac{S^*}{S} - \frac{E}{E^*} - \frac{\eta _{s}^{\alpha } S E^*}{(\eta _{s}^{\alpha })^* S^* E} + \frac{\eta _{s}^{\alpha }}{(\eta _{s}^{\alpha })^*}\right) + a(\eta _{s}^{\alpha })^*V^* \left( 2 - \frac{V^*}{V} - \frac{E}{E^*} - \frac{\eta _{s}^{\alpha } V E^*}{(\eta _{s}^{\alpha })^* V^* E} + \frac{\eta _{s}^{\alpha }}{(\eta _{s}^{\alpha })^*}\right) \\&\quad + \frac{\sigma ^{\alpha } \tau ^{\alpha } (\eta _{s}^{\alpha })^{*} S^{*} E^{*}}{(\sigma ^{\alpha } \tau ^{\alpha } E^{*} + \gamma _{1}^{\alpha } I_{a}^{*})} \left( \frac{E}{E^*} - \frac{I_{s}}{I_{s}^*} - \frac{E I_{s}^*}{E^* I_{s}} + 1\right) + \frac{\gamma _{1}^{\alpha } (\eta _{s}^{\alpha })^{*} S^{*} I_{a}^{*}}{(\sigma ^{\alpha } \tau ^{\alpha } E^{*} + \gamma _{1}^{\alpha } I_{a}^{*})} \left( \frac{I_{a}}{I_{a}^*} - \frac{I_{s}}{I_{s}^*} - \frac{I_{a} I_{s}^*}{I_{a}^* I_{s}} + 1\right) \\&\quad + \frac{(\eta _{s}^{\alpha })^{*} E^{*} I_{a}^{*} G_{1}}{G_{2}} \left( \frac{E}{E^*} - \frac{I_{a}}{I_{a}^*} - \frac{E I_{a}^*}{E^* I_{a}} + 1\right) + \frac{a \tau ^{\alpha } k_{6} (\eta _{s}^{\alpha })^{*} V^{*}}{(\tau ^{\alpha } k_{6} E^{*} + \gamma _{2}^{\alpha } I_{a}^{*})} \left( \frac{E}{E^*} - \frac{I_{m}}{I_{m}^*} - \frac{E I_{m}^*}{E^* I_{m}} + 1\right) \\&\quad + \frac{a \gamma _{2}^{\alpha } (\eta _{s}^{\alpha })^{*} V^{*} I_{a}^{*}}{(\tau ^{\alpha } k_{6} E^{*} + \gamma _{2}^{\alpha } I_{a}^{*})} \left( \frac{I_{a}}{I_{a}^*} - \frac{I_{m}}{I_{m}^*} - \frac{I_{a} I_{m}^*}{I_{a}^* I_{m}} + 1\right) . \end{aligned}$$

Following the idea in^73,77^, suppose we have a function defined as $\chi (x) = 1 - x + \ln (x),$ if $x > 0$, we have $\chi (x) \le 0,$ and if $x = 1,$ we have $\chi (1) = 0. \text { Thus }, x - 1 \ge \ln (x) \text { for } x >0$ Using this relation we find that

A-9 $$\begin{aligned} \left( 2 - \frac{S^*}{S} - \frac{E}{E^*} - \frac{\eta _{s}^{\alpha } S E^*}{(\eta _{s}^{\alpha })^* S^* E} + \frac{(\eta _{s}^{\alpha })}{(\eta _{s}^{\alpha })^*}\right)&= -\left( 1 - \frac{\eta _{s}^{\alpha }}{(\eta _{s}^{\alpha })^*}\right) \left( 1 - \frac{I_{s} (\eta _{s}^{\alpha })^*}{I_{s}^*\eta _{s}^{\alpha }}\right) \\&\quad + 3 - \frac{S^*}{S} - \frac{\eta _{s}^{\alpha } S E^*}{(\eta _{s}^{\alpha })^* S^* E} - \frac{I_{s} (\eta _{s}^{\alpha })^*}{I_{s}^* \eta _{s}^{\alpha }} - \frac{E}{E^*} + \frac{I_{s}}{I_{s}^*} \\&\le -\left( \frac{S^*}{S} - 1\right) - \left( \frac{\eta _{s}^{\alpha } S E^*}{(\eta _{s}^{\alpha })^* S^* E} - 1\right) - \left( \frac{I_{s} (\eta _{s}^{\alpha })^*}{I_{s}^* \eta _{s}^{\alpha }} - 1 \right) - \frac{E}{E^*} + \frac{I_{s}}{I_{s}^*} \\&\le -\ln \left( \frac{S^* \eta _{s}^{\alpha } S E^* I_{s} (\eta _{s}^{\alpha })^*}{S (\eta _{s}^{\alpha })^* S^* E I_{s}^* \eta _{s}^{\alpha }}\right) - \frac{E}{E^*} + \frac{I_{s}}{I_{s}^*} = \frac{I_{s}}{I_{s}^*} - \ln \left( \frac{I_{s}}{I_{s}^*}\right) + \ln \left( \frac{E}{E^*}\right) - \frac{E}{E^*}. \end{aligned}$$

Likewise,

A-10 $$\begin{aligned} \left( 2 - \frac{V^*}{S} - \frac{E}{E^*} - \frac{\eta _{s}^{\alpha } V E^*}{(\eta _{s}^{\alpha })^* V^* E} + \frac{\eta _{s}^{\alpha }}{(\eta _{s}^{\alpha })^*}\right)&= -\left( 1 - \frac{\eta _{s}^{\alpha }}{(\eta _{s}^{\alpha })^*}\right) \left( 1 - \frac{I_{m} (\eta _{s}^{\alpha })^*}{I_{m}^*\eta _{s}^{\alpha }}\right) \\&\quad + 3 - \frac{V^*}{V} - \frac{\eta _{s}^{\alpha } V E^*}{(\eta _{s}^{\alpha })^* V^* E} - \frac{I_{m} (\eta _{s}^{\alpha })^*}{I_{m}^* \eta _{s}^{\alpha }} - \frac{E}{E^*} + \frac{I_{m}}{I_{m}^*} \\&\le -\left( \frac{V^*}{V} - 1\right) - \left( \frac{\eta _{s}^{\alpha } V E^*}{(\eta _{s}^{\alpha })^* V^* E} - 1\right) - \left( \frac{I_{m} (\eta _{s}^{\alpha })^*}{I_{m}^* \eta _{s}^{\alpha }} - 1 \right) - \frac{E}{E^*} + \frac{I_{m}}{I_{m}^*} \\&\le -\ln \left( \frac{V^* \eta _{s}^{\alpha } V E^* I_{m} (\eta _{s}^{\alpha })^*}{V (\eta _{s}^{\alpha })^* V^* E I_{m}^* \eta _{s}^{\alpha }}\right) - \frac{E}{E^*} + \frac{I_{m}}{I_{m}^*} = \frac{I_{m}}{I_{m}^*} - \ln \left( \frac{I_{m}}{I_{m}^*}\right) + \ln \left( \frac{E}{E^*}\right) - \frac{E}{E^*}. \end{aligned}$$

Meanwhile, one can still verify that

A-11 $$\begin{aligned} \left( \frac{E}{E^*} - \frac{I_{s}}{I_{s}^*} - \frac{I_{s}^*E}{I_{s}E^*} + 1 \right)&= -\left( \frac{I_{s}^*E}{I_{s}E*} - 1\right) + \frac{E}{E*} - \frac{I_{s}}{I_{s}*} \le -\ln \left( \frac{I_{s}^*E}{I_{s}E*}\right) + \frac{E}{E*} - \frac{I_{s}}{I_{s}*} \\&\le - \ln \left( \frac{I_{s}^*}{I_{s}}\right) - \ln \left( \frac{E}{E*}\right) + \frac{E}{E*} - \frac{I_{s}}{I_{s}*} = \frac{E}{E*} - \ln \left( \frac{E}{E*}\right) - \frac{I_{s}}{I_{s}*} + \ln \left( \frac{I_{s}}{I_{s}*}\right) . \end{aligned}$$

Similarly, we have

A-12 $$\begin{aligned} \left( \frac{I_{a}}{I_{a}^*} - \frac{I_{s}}{I_{s}^*} - \frac{I_{s}^*I_{a}}{I_{s}I_{a}^*} + 1 \right) \le \frac{I_{a}}{I_{a}*} - \ln \left( \frac{I_{a}}{I_{a}*}\right) - \frac{I_{s}}{I_{s}*} + \ln \left( \frac{I_{s}}{I_{s}*}\right) , \end{aligned}$$

A-13 $$\begin{aligned} \left( \frac{E}{E^*} - \frac{I_{a}}{I_{a}^*} - \frac{I_{a}^*E}{I_{a}E^*} + 1 \right) \le \frac{E}{E*} - \ln \left( \frac{E}{E*}\right) - \frac{I_{a}}{I_{a}*} + \ln \left( \frac{I_{a}}{I_{a}*}\right) , \end{aligned}$$

A-14 $$\begin{aligned} \left( \frac{E}{E^*} - \frac{I_{m}}{I_{m}^*} - \frac{I_{m}^*E}{I_{m}E^*} + 1 \right) \le \frac{E}{E*} - \ln \left( \frac{E}{E*}\right) - \frac{I_{m}}{I_{m}*} + \ln \left( \frac{I_{m}}{I_{m}*}\right) , \end{aligned}$$

and

A-15 $$\begin{aligned} \left( \frac{I_{a}}{I_{a}^*} - \frac{I_{m}}{I_{m}^*} - \frac{I_{m}^*I_{a}}{I_{m}I_{a}^*} + 1 \right) \le \frac{I_{a}}{I_{a}*} - \ln \left( \frac{I_{a}}{I_{a}*}\right) - \frac{I_{m}}{I_{m}*} + \ln \left( \frac{I_{m}}{I_{m}*}\right) . \end{aligned}$$

Substituting (A-9)–(A-15) in (A-8) we have,

A-16 $$\begin{aligned} \dot{F_{2}}(t)&\le (\eta _{s}^{\alpha })^*S^* \left( \frac{I_{s}}{I_{s}^*} - \ln \left( \frac{I_{s}}{I_{s}^*}\right) + \ln \left( \frac{E}{E^*}\right) - \frac{E}{E^*}\right) \\&\quad + a(\eta _{s}^{\alpha })^*V^* \left( \frac{I_{m}}{I_{m}^*} - \ln \left( \frac{I_{m}}{I_{m}^*}\right) + \ln \left( \frac{E}{E^*}\right) - \frac{E}{E^*}\right) \\&\quad + \frac{\sigma ^{\alpha } \tau ^{\alpha } (\eta _{s}^{\alpha })^{*} S^{*} E^{*}}{(\sigma ^{\alpha } \tau ^{\alpha } E^{*} + \gamma _{1}^{\alpha } I_{a}^{*})} \left( \frac{E}{E*} - \ln \left( \frac{E}{E*}\right) + \ln \left( \frac{I_{s}}{I_{s}*}\right) - \frac{I_{s}}{I_{s}*}\right) \\&\quad + \frac{\gamma _{1}^{\alpha } (\eta _{s}^{\alpha })^{*} S^{*} I_{a}^{*}}{(\sigma ^{\alpha } \tau ^{\alpha } E^{*} + \gamma _{1}^{\alpha } I_{a}^{*})} \left( \frac{I_{a}}{I_{a}*} - \ln \left( \frac{I_{a}}{I_{a}*}\right) + \ln \left( \frac{I_{s}}{I_{s}*}\right) - \frac{I_{s}}{I_{s}*}\right) \\&\quad + \frac{(\eta _{s}^{\alpha })^{*} E^{*} I_{a}^{*} G_{1}}{G_{2}} \left( \frac{E}{E*} - \ln \left( \frac{E}{E*}\right) + \ln \left( \frac{I_{a}}{I_{a}*}\right) - \frac{I_{a}}{I_{a}*}\right) \\&\quad + \frac{a \tau ^{\alpha } k_{6} (\eta _{s}^{\alpha })^{*} V^{*}}{(\tau ^{\alpha } k_{6} E^{*} + \gamma _{2}^{\alpha } I_{a}^{*})} \left( \frac{E}{E*} - \ln \left( \frac{E}{E*}\right) + \ln \left( \frac{I_{m}}{I_{m}*}\right) - \frac{I_{m}}{I_{m}*} \right) \\&\quad + \frac{a \gamma _{2}^{\alpha } (\eta _{s}^{\alpha })^{*} V^{*} I_{a}^{*}}{(\tau ^{\alpha } k_{6} E^{*} + \gamma _{2}^{\alpha } I_{a}^{*})} \left( \frac{I_{a}}{I_{a}*} - \ln \left( \frac{I_{a}}{I_{a}*}\right) + \ln \left( \frac{I_{m}}{I_{m}*}\right) - \frac{I_{m}}{I_{m}*}\right) . \end{aligned}$$

Hence (A-2)–(A-16) ensure that $\frac{dF_{2}}{dt} \le 0$. It is easy to see that $\frac{dF_{2}}{dt} = 0$ holds only for $S = S^*, V = V^*, E = E^*, I_{s} = I_{s}^*, I_{a} = I_{a}^*, I_{m} = I_{m}^*, \text { and } R = R^*$. In a similar manner in^78^, every solution of our model system (11) with initial conditions in ${\mathbb {D}}$ approaches the stable *EE* as $t \longrightarrow \infty$. Hence, *EE* is GAS equilibrium of (11) on ${\mathbb {D}}$. $\square$
